# Targeting mTORC2/HDAC3 Inhibits Stemness of Liver Cancer Cells Against Glutamine Starvation

**DOI:** 10.1002/advs.202103887

**Published:** 2022-02-20

**Authors:** Hui‐Lu Zhang, Ping Chen, He‐Xin Yan, Gong‐Bo Fu, Fei‐Fei Luo, Jun Zhang, Shi‐Min Zhao, Bo Zhai, Jiang‐Hong Yu, Lin Chen, Hao‐Shu Cui, Jian Chen, Shuai Huang, Jun Zeng, Wei Xu, Hong‐Yang Wang, Jie Liu

**Affiliations:** ^1^ Department of Digestive Diseases of Huashan Hospital and Institutes of Biomedical Sciences Fudan University Shanghai 200040 China; ^2^ Renji Hospital School of Medicine Shanghai Jiaotong University Shanghai 200120 China; ^3^ Department of Medical Oncology Affiliated Jinling Hospital Medical School of Nanjing University Nanjing 210093 China; ^4^ Key Laboratory of Separation Science for Analytical Chemistry Dalian Institute of Chemical Physics Chinese Academy of Sciences Dalian 116023 China; ^5^ Eastern Hepatobiliary Surgery Hospital Second Military Medical University Shanghai 200433 China; ^6^ National Center for Liver Cancer Shanghai 200433 China

**Keywords:** glutamine starvation, glutamine synthetase, HDAC3, Rictor/mTORC2, tumor initiating cells

## Abstract

Cancer cells are addicted to glutamine. However, cancer cells often suffer from glutamine starvation, which largely results from the fast growth of cancer cells and the insufficient vascularization in the interior of cancer tissues. Herein, based on clinical samples, patient‐derived cells (PDCs), and cell lines, it is found that liver cancer cells display stem‐like characteristics upon glutamine shortage due to maintaining the stemness of tumor initiating cells (TICs) and even promoting transformation of non‐TICs into stem‐like cells by glutamine starvation. Increased expression of glutamine synthetase (GS) is essential for maintaining and promoting stem‐like characteristics of liver cancer cells during glutamine starvation. Mechanistically, glutamine starvation activates Rictor/mTORC2 to induce HDAC3‐mediated deacetylation and stabilization of GS. Rictor is significantly correlated with the expression of GS and stem marker OCT4 at tumor site, and closely correlates with poor prognosis of hepatocellular carcinomas. Inhibiting components of mTORC2‐HDAC3‐GS axis decrease TICs and promote xenografts regression upon glutamine‐starvation therapy. Collectively, the data provides novel insights into the role of Rictor/mTORC2‐HDAC3 in reprogramming glutamine metabolism to sustain stemness of cancer cells. Targeting Rictor/HDAC3 may enhance the efficacy of glutamine‐starvation therapy and limit the rapid growth and malignant progression of tumors.

## Graphical abstract

Cancer cells are addicted to glutamine. When glutamine is deficient, mTORC1 is inhibited but mTORC2 is activated to regulate metabolic balance. Rictor/mTORC2 activates histone deacetylase HDAC3, which stabilizes glutamine synthase (GS) expression. GS promotes endogenous glutamine synthesis and maintains intracellular glutamine balance, which endows cancer cells with stem cell characteristics and promotes the malignant progression of cancer.

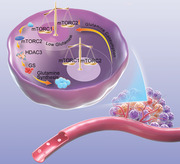

## 1 Introduction

Metabolic reprogramming is important for malignant progression and tumorigenesis.^[^ ^1^, ^2^ ^]^ Growing evidence suggests that oncogenic reprogramming of glutamine metabolism to the tricarboxylic acid (TCA) cycle, a process catalyzed by glutaminase (GLS) and named glutaminolysis, supports cancer cell proliferation.^[^ ^3^, ^4^ ^]^ This is due at least in part to glutamine being essential for the synthesis of nucleotides and nonessential amino acids during cancer development.^[^ ^5^ ^]^ Several cancers have been found to display increased glutamine uptake and glutaminolysis, rendering cells addicted to glutamine.^[^ ^3^, ^4^ ^]^ Glutaminolysis inhibitors, such as BPTES and Compound 968, have been developed as anticancer therapies.^[^ ^6^, ^7^, ^8^ ^]^

Glutamine can be taken up from environment as well as synthesized from *α*‐ketoglutarate by glutamine synthetase (GS). Latest studies have shown that increased glutamine synthesis upon GS overexpression, rather than glutaminolysis, also promotes tumor growth and progression by enhancing nucleotide synthesis and amino transport.^[^ ^5^, ^9^ ^]^ These results indicate that glutamine anabolism and catabolism both play roles in cancer development. In fact, due to fast growth of cancer cells and the insufficient vascularization in the interior of cancer tissues, exogenous glutamine is limited in the tumor microenvironment.^[^ ^10^ ^]^ However, it is unclear how cancer cells perceive the lack of exogenous glutamine and regulate the synthesis of endogenous glutamine. GS has been reported to play an important role in regulating intracellular glutamine balance and anabolism under exogenous glutamine deficiency.^[^ ^9^ ^]^ When glutamine is sufficient, acetylation can promote GS degradation and reduce unnecessary endogenous glutamine synthesis.^[^ ^11^, ^12^ ^]^ However, it is not clear how to initiate endogenous glutamine synthesis by stabilizing GS expression when glutamine is deficient.

Tumor heterogeneity is thought to be a result of the hierarchical organization of tumor cells by a subset of cells with stem cell features known as tumor initiating cells (TICs).^[^ ^13^, ^14^, ^15^ ^]^ The stemness of cancer cells has been reported as being responsible for the initiation and progression of tumors in a variety of cancers.^[^ ^15^, ^16^ ^]^ Targeting TICs is therefore a promising approach to eradicating the tumor and improving poor prognosis in cancer patients. Despite advances in the understanding of molecular signaling for the regulation of TICs, effective therapies to eradicate TICs have had limited success. Recent studies showed that ASCT2‐mediate uptake of exogenous glutamine can maintain TICs self‐renewal in pancreatic cancer^[^ ^17^ ^]^ and exogenous glutamine can maintain TICs self‐renewal by a redox‐mediated mechanism mediated by *β*‐catenin in non‐small cell lung carcinoma and pancreatic cancer.^[^ ^18^ ^]^ These studies indicate that the survival and self‐renewal of tumor initiating cells depend on glutamine. However, it is not clear how tumor initiating cells regulate their own survival and self‐renewal in the glutamine deficient tumor microenvironment.

In the present study, we found that liver cancer cells displayed stem‐like characteristics upon glutamine deprivation by the maintain of stemness in TICs and the transformation of non‐TICs into TICs. From the mechanical point of view, glutamine deprivation activated mTORC2 to promote HDAC3‐mediated deacetylation and stabilization of GS, which involved in the supporting stemness of cancer cells. These findings supply a novel mechanism for the resistance of liver cancer cell to glutamine deprivation, and also identify mTORC2 and GS as the promising prognosis biomarkers for hepatocellular carcinomas.

## 2 Results

### 2.1 Glutamine Starvation Promotes Stemness of Liver Cancer Cells

**Figure 1:**
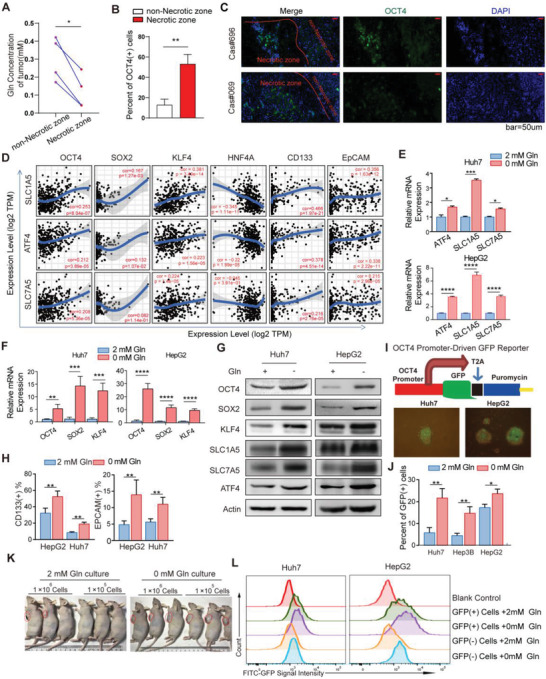
Glutamine starvation promotes stemness of liver cancer cells A) Quantification of glutamine concentration in the core zones with more necrotic tissue areas and in the periphery zones with no necrotic tissue areas of fresh tumors from HCC patients (*n* = 4). Gln, glutamine. B) The proportion of OCT4 positive cells detected by flow cytometry (FC) in living cells from the core zones with more necrotic tissue areas and the periphery zones with no necrotic tissue areas of fresh tumors from HCC patients (*n* = 3). C) Representative immunohistochemistry (IHC) and immunofluorescence (IF) images of OCT4 positive cells in nutrient limited tumors from HCC patients. Scale bars, 25 µm. D) The correlation between ATF4, SLC1A5 or SLC7A5 and OCT4, SOX2, KLF4, CD133, and EpCAM expression in liver hepatocellular carcinoma (LIHC) data from The Cancer Genome Atlas (TCGA) cohort. Spearman's correlation was used to assess the relationship between genes. E,F) The mRNA relative expression of ATF4, SLC1A5, SLC7A5, OCT4, SOX2, and KLF4 in Huh7 and HepG2 cell lines after glutamine starvation for 48 h, in contrast to normal culture medium (*n* = 4). G) The protein expression of OCT4, SOX2, KLF4, ATF4, SLC1A5, and SLC7A5 in Huh7 and HepG2 cell lines after glutamine starvation for 48 h, in contrast to normal culture medium. H) Percent of CD133 (+) cells and EpCAM (+) cells detected by FC in Huh7 and HepG2 cells cultured in medium with or without glutamine for 48 h (*n* = 6). I) Structural diagram of OCT4 promoter‐driven GFP plasmid based on the lentiviral vector; representative photographs showing sphere formation capacity of Huh7 and HepG2 cell lines on day 12; representative images of OCT4‐GFP (+) cells in Huh7 and HepG2 cell lines cultured in medium with or without glutamine for 48 h. J) Percent of GFP (+) cells detected by FC in various cancer cell lines cultured in medium with or without glutamine for 48 h (*n* = 3). (K) Huh7 cells were cultured in medium with (2 × 10^−3^ m) or without glutamine for 48 h and then inoculated different doses (1 × 10^6^ or 1 × 10^5^ cells) in nude mice. Representative photographs show the tumor volume difference. (L) OCT4‐GFP (+) and OCT4‐GFP (−) cells were selected by FC and cultured with or without glutamine for 48 h. The average fluorescence intensity of GFP were detected by FITC channel of FC (*n* = 3). All data are shown in A,B, E,F, J) as the mean values ± SD, *p* values are based on Student's *t* test. ****p < 0.0001, ***p < 0.001, **p < 0.01, *p < 0.05.

To elucidate the response of liver tumor initiating cells to glutamine shortage, we analyzed the fresh tumor samples from HCC patients. As shown in Figure S1A (Supporting Information), we cut 5 × 5 mm size tissue in the necrotic tissue area (the area marked “*a*”) and the same size tissue in non‐necrotic area (the area marked “*b*”). Then we sorted the primary cells respectively, and analyzed the proportion of necrotic cells in different tissue areas by flow cytometry. Propidium iodide (PI) staining confirmed that the collected tissues in necrotic areas contained more dead cells than those in non‐necrotic areas (Figure S1B, Supporting Information). As in line with previous reports,^[^ ^10^ ^]^ the concentration of glutamine was much lower in the core zones with more necrotic tissue areas than that in the periphery zones with no necrotic tissue areas (**Figure** **1**A), thus the core zones were regarded as the glutamine shortage zones. Notably, most of the live cells in the glutamine shortage zones of tumors from HCC patients expressed OCT4 (Figure 1B,C; Figure S1C, Supporting Information), a core pluripotent transcription factor in stem cells and is known to drive self‐renewal of human stem‐like cells.^[^ ^19^, ^20^, ^21^, ^22^ ^]^ Meanwhile, liver hepatocellular carcinoma (LIHC) data from The Cancer Genome Atlas (TCGA) cohort showed that the expression of stem genes OCT4, SOX2, KLF4, CD133, EPCAM, and differentiation related gene HNF4A were correlated with several glutamine shortage‐sensitive genes, SLC1A5, SLC7A5, and ATF4^[^ ^23^ ^]^ (Figure 1D). To further confirm the above observation, two kinds of liver cancer cell lines, Huh7 and HepG2, were treated with or without 2 × 10^−3^ m glutamine. Two days later, cells in glutamine deprivation groups showed higher mRNA and protein levels of core functional markers of stem cells (OCT4, SOX2, and KLF4) and also glutamine‐sensitive genes (ATF4, SLC1A5, and SLC7A5) than those in control groups (Figure 1E–G). CD133 and EpCAM have been regarded as surface markers of tumor initiating cells or tumor‐initiating cells in liver cancer.^[^ ^24^, ^25^, ^26^ ^]^ Consistently, both proportions of CD133 and EpCAM were significantly increased in Huh7 and HepG2 cell lines, and patient‐derived cells (PDCs) in response to glutamine deprivation for 2 days (Figure 1H; Figure S1D, Supporting Information). Previous studies have reported that OCT4 activation drove self‐renewal of human TICs.^[^ ^19^, ^20^, ^21^ ^]^ Our study also demonstrated that OCT4 was required for maintaining liver TICs in HCCs.^[^ ^22^ ^]^ So, we established liver cancer cell lines in which OCT4 promoter drives GFP expression (Figure 1I). Following glutamine deprivation, the percentage of OCT4 expression reported by GFP was greatly increased in both Huh7, Hep3B and HepG2 liver cancer cell lines (Figure 1J). More importantly, Huh7 cells upon glutamine starvation had enhanced capacity to form xenograft in mice (Figure 1K; Figure S1E, Supporting Information). These data suggest that glutamine starvation enriches the stem‐like characteristics of liver cancer cells.

Next, we planned to identify the enhanced stem‐cell characteristics of tumor cells upon glutamine deficiency due to the stronger proliferative ability of TICs or the transformation of non‐TICs into TICs. OCT4‐GFP (+) cells and OCT4‐GFP (−) cells represented for TICs and non‐TICs respectively, and were separately sorted by flow cytometry for CCK‐8 assay to detect the proliferative ability (Figure S2A, Supporting Information). The proliferation of GFP (+) cells was similar to that of GFP (−) cells (Figure S2B, Supporting Information), but the proportion of GFP (+) cells was increased much faster than that of GFP (−) cells in the absence of glutamine for 48 h (Figure 1J). Meanwhile, we also tested whether glutamine deficiency promoted the transformation of nonstem cells into stem‐like cells. GFP (+) cells and GFP (−) cells were separately sorted and cultured with or without glutamine. After 48 h, the fluorescence intensity of GFP driven by OCT4 promoter was significantly enhanced in GFP (−) cell groups under glutamine deficiency condition (Figure 1L; Figure S2C,D, Supporting Information). Consistently, real‐time PCR results showed that glutamine deficiency promoted OCT4 expression both in OCT4‐GFP (+) cells and OCT4‐GFP (−) cells (Figure S2E, Supporting Information). These results suggest that glutamine deficiency not only maintains the stem cell characteristics of TICs, but also promotes the transformation of non‐TICs into stem‐like cells.

### 2.2 Liver Cancer Cells Upregulate Glutamine Synthetase Expression to Sustain Stem‐Like Characteristics upon Glutamine Starvation

**Figure 2:**
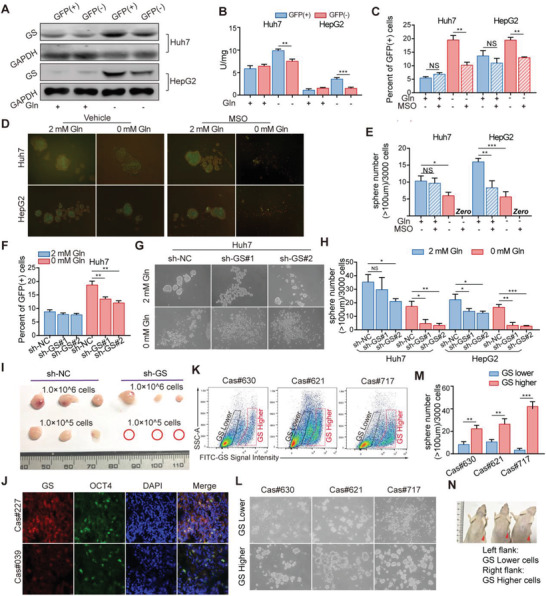
A,B) Liver cancer cells upregulate glutamine synthetase expression to sustain stem‐like characteristics upon glutamine starvation A) GS expression and B) activity were determined in GFP (+) or GFP (−) cells which were selected by fluorescence‐activated cell sorting (FACS) from Huh7 and HepG2 cells cultured in medium with or without glutamine for 12 h. C) Percent of OCT4‐GFP (+) cells detected by FC in Huh7 and HepG2 cells pretreated with MSO (1 × 10^−3^ m) and cultured in medium with or without glutamine for 48 h (*n* = 3). D) Representative images and E) quantification of the sphere numbers in Huh7 and HepG2 cells with treatment of MSO (1 × 10^−3^ m) in medium with or without glutamine (*n* = 6). F) Percent of GFP (+) cells detected by FC in Huh7 cells expressing sh‐NC or sh‐GS cultured in medium with or without glutamine for 48 h (*n* = 3). G) Representative images and H) quantification of the sphere numbers of Huh7 and HepG2 cells expressing sh‐Con or sh‐GS cultured in medium with or without glutamine for 12 days (*n* = 6). I) Representative images of xenograft tumors injected with 1.0 × 10^6^ and 1.0 × 10^5^ Huh7 cells expressing sh‐NC and sh‐GS in NSG mice (*n* = 3). The tumor‐harboring mice were sacrificed on day 14 after the first measurement. J) Representative images of GS and OCT4 expression in human liver cancer samples by immunofluorescence (IF) assay. Scale bars, 10 µm. K) Representative FC plots showing FITC‐GS staining levels in primary patient liver cancer cells. L) Representative images and M) quantification of the sphere numbers in GS high and low expression cells selected by FC from primary patient liver cancer cells treated with Gln‐free medium without serum for 12 days. N) Representative images of PDX tumors formed by cells with GS lower (Left flank) and higher (Right flank) expression which were selected by FC from primary patient liver cancer cells. All data are shown as the mean values ± SD, *p* values are based on Student's *t* test. ***p < 0.001, **p < 0.01, *p < 0.05; NS, nonsignificant.

Considering the essential role of glutamine for the survival of tumor cells, we sought for the alternative pathway for glutamine supply in tumor cells upon exogenous glutamine shortage. We found that the broad range of glutamine‐requiring enzymes including GLS and nucleotides synthetases inhibitor 6‐diazo‐5‐oxo‐l‐norleucine (DON),^[^ ^27^, ^28^ ^]^ but not GLS inhibitor bis‐2‐(5‐phenylacetamido‐1,3,4‐thiadiazol‐2‐yl) ethyl sulfide (BPTES), significantly inhibited the formation of spheres in both Huh7 and HepG2 cells following glutamine shortage (Figure S3A,B, Supporting Information). More importantly, glutamine withdrawal‐induced OCT4^+^ Huh7 and HepG2 cells exhibited elevated anabolic pathway‐involved glutamine synthetase (GS) expression and activity compared with OCT4^–^ cells upon glutamine shortage (**Figure** **2**A,B). Notably, only withdrawal of glutamine among all proteinogenic amino acids induced GS overexpression in vitro (Figure S3C, Supporting Information). Then, GS inhibitor l‐Mmethionine sulfoximine (MSO) abolished the increased percentage of OCT4^+^ cells induced by glutamine deprivation in Huh7 and HepG2 cells, and even completely prevented sphere formation following glutamine withdrawal in liver cancer cell lines and primary liver cancer cells (Figure 2C–E and Figure S4A,B, Supporting Information). Moreover, GS knock‐down abolished the increased percentage of OCT4^+^ cells induced by glutamine deprivation (Figure 2F). Notably, GS deficiency greatly reduced frequency of spheroids initiating cells and spheroids formation in both Huh7 and HepG2 cells in response to the absence of glutamine (Figure 2G,H; Figure S5A,B, Supporting Information). Knock‐down of GS in Huh7 cells further led to retarded and reduced xenograft tumor formation and tumorigenic cell frequency in NOD acid gamma (NSG) mice (Figure 2I; Figure S5D, Supporting Information). Thus, the maintenance of the characteristics of liver TICs under glutamine starvation mainly depends on anabolic pathway.

Moreover, we confirmed the above results in both primary cancer cells and tissues of patients. As shown in Figure S4C (Supporting Information), GS expression was significantly higher in patient‐derived xenograft (PDX) models after tumor formation compared with the primary cancer tissues. Also, patients‐derived OCT4, CD133 or EpCAM positive primary liver cancer cells displayed higher levels of GS expression than those negative cells (Figure 2J; Figure S4D, Supporting Information). Then, these primary patient liver cancer cells were sorted into two groups based on GS high or low expression (Figure 2K). Primary cancer cells with high expression of GS had stronger ability to form spheres than those with low expression of GS when cultured in glutamine‐deprived media (Figure 2L,M). More importantly, primary liver cancer cells in high GS group showed much stronger potency to form PDX than those in low GS group within the same sample (Figure 2N). These results show that the relative lack of exogenous glutamine in the process of tumor formation induces the increase of GS expression, and GS is critical essential to support the properties of liver TICs and tumor formation.

### 2.3 GS Mediated Nucleotide Synthesis Sustains the Stem‐Like Characteristics of Cancer Cells in Glutamine Deficiency

**Figure 3:**
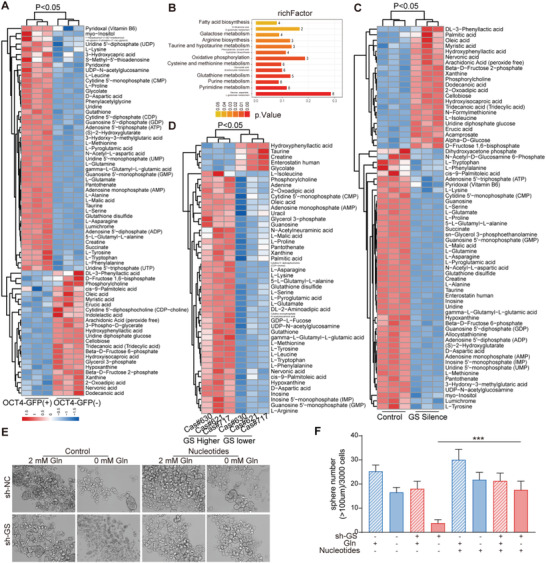
GS mediated nucleotide synthesis sustains the stem‐like characteristics of cancer cells in glutamine deficiency. A,B) Metabolite cluster gram and metabolic pathway enrichment analysis of OCT4‐GFP (+) and OCT4‐GFP (−) cells selected by FACS from Huh7 cells, cultured in medium without glutamine for 12 h (*n* = 3). C) Metabolite cluster gram and metabolic pathway enrichment analysis of GFP (+) cells selected by FACS from control and GS knock‐down Huh7 cells, cultured in medium without glutamine for 12 h (*n* = 3). D) Metabolite cluster gram and metabolic pathway enrichment analysis of GS higher and lower expression cells selected by FACS from primary patient liver cancer cells. E) Sphere formation assay of control and GS knock‐down Huh7 cells in culture medium with supplement of glutamine (2 × 10^−3^ m) or nucleotides (containing AGCTU, 0.2 × 10^−3^ m each). Spheres (diameter > 100 µm) counted on day 12 (*n* = 6). The significant different metabolites were determined based on the combination of a statistically significant threshold of variable influence on projection (VIP) values obtained from PLS‐DA model and two‐tailed Student's *t* test (*p* value) on the raw data, and the metabolites with VIP values larger than 1.0 and p values less than 0.1 were considered as significant. The data are shown in (F) as the mean values ± SD, *p* values are based on Student's *t* test. ***p < 0.001.

To investigate how GS sustained the stem‐like characteristics of cancer cells upon microenvironmental glutamine deficiency, we compared the steady state metabolites levels between OCT4^+^ and OCT4^–^ cancer cells. Higher levels of nucleotides and l‐glutamine were detected in OCT4^+^ cancer cells compared to those in OCT4^–^ cancer cells when cultured in glutamine‐limiting media (**Figure** **3**A,B). Moreover, the nucleotides levels were decreased in GS knock‐down OCT4^+^ cancer cells (Figure 3C). Primary liver cancer cells with higher GS expression had higher levels of nucleotides than those with lower GS expression (Figure 3D). Furthermore, nucleotides supplement rescued the decrease sphere formation of GS silenced cells cultured in glutamine‐limiting media (Figure 3E,F). Collectively, liver cancer cells upregulated GS expression to supply glutamine and nucleotides, and thus maintain stem‐like characteristics upon glutamine starvation.

### 2.4 Rictor/mTORC2 but not mTORC1 is Required for Sustaining GS Expression and Stemness of Liver Cancer Cells upon Glutamine Starvation

**Figure 4:**
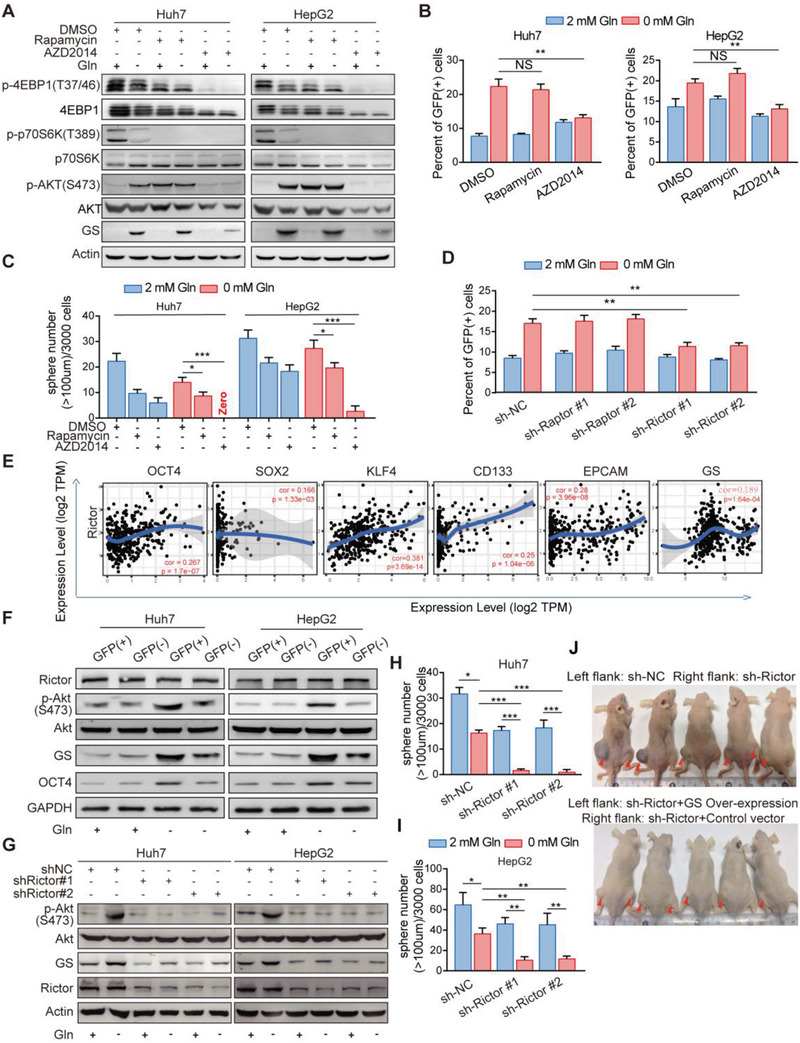
Activation of Rictor/mTORC2 is required for GS upregulation in response to glutamine starvation A) Immunoblot analysis of GS, phosphorylation of AKT (S473), 4EBP1(T47/36), and p70S6K (T389) expression in Huh7 and HepG2 cells with treatment of DMSO, Rapamycin (100 ×10^−9^ m) or AZD2014 (2 × 10^−6^ m) cultured in medium with or without glutamine for 12 h. B) Percent of OCT4‐GFP (+) cells detected by FC in HepG2 and Huh7 cells pretreated with DMSO, Rapamycin (100 × 10^−9^ m), AZD2014 (2 × 10^−6^ m) cultured in medium with or without glutamine for 48 h (*n* = 3). C) Quantification of the sphere numbers (D) of Huh7 and HepG2 cells with treatment of DMSO, Rapamycin (100 × 10^−9^ m) or AZD2014 (2 × 10^−6^ m), cultured in medium with or without glutamine for 12 days (*n* = 6). Scale bar, 100 mm. D) Percent of OCT4‐GFP (+) cells detected by FC in Huh7 cells expressing sh‐NC, sh‐Rap or sh‐Ric cultured in medium with or without glutamine for 48 h (*n* = 6). Con, Rap, and Ric are short for control, Raptor and Rictor, respectively. E) The correlation between ATF4, SLC1A5, or SLC7A5, and OCT4 expression level in liver hepatocellular carcinoma (LIHC) data from The Cancer Genome Atlas (TCGA) cohort. Spearman's correlation was used to assess the relationship between genes. F) Immunoblot analysis for the indicated proteins from GFP (+) and GFP (−) cells isolated by FACS from Huh7 and HepG2 cells cultured in medium with or without glutamine for 12 h. G) Immunoblot analysis of GS in control or Rictor knock‐down Huh7 and HepG2 cells cultured in medium with or without glutamine for 12 h. H,I) Quantification of the sphere numbers (J) of Huh7 and HepG2 cells expressing sh‐NC or sh‐Rictor cultured in medium with or without glutamine for 12 days (*n* = 6). NC, negative control. J) Representative images of xenograft models established by injecting 1.0 × 10^6^ Huh7 cells expressing sh‐NC (Left flank, upper), sh‐Rictor (Right flank, upper), sh‐Rictor and constitutive GS (Left flank, bottom) or sh‐Rictor and control vector (Right flank, bottom) in nude mice. The tumor‐harboring mice were sacrificed in day 15 after the first measurement (*n* = 5). All data are shown in (B–D, H–I) as the mean values ± SD, *p* values are based on Student's *t* test. ***p < 0.001, **p < 0.01, *p < 0.05; NS, nonsignificant.

Given the mTOR as the master sensor of glutamine,^[^ ^29^, ^30^ ^]^ we planned to investigate whether glutamine shortage could induce GS expression via activation of mTORC1/2. When mTORC1 was inhibited with rapamycin, glutamine starvation still effectively induced GS upregulation in both Huh7 and HepG2 cells; however, when both mTORC1 and mTORC2 were simultaneously inhibited by AZD2014, glutamine starvation‐induced GS upregulation was abolished (**Figure** **4**A). This notion was further substantiated by treatment with AZD2014, but not rapamycin, which efficiently prevented the increased expression of OCT4 and inhibited the enhanced ability of sphere formation in both Huh7 and HepG2 cells following glutamine deprivation (Figure 4B,C). These findings suggest that mTORC2 is involved in glutamine deprivation‐induced GS upregulation. Next, in order to further confirm the role of mTORC2 in the regulation of GS expression, we knocked down mTORC1 signature partner Raptor or mTORC2 signature partner Rictor. As shown in Figure 4D, only Rictor deficiency abolished the increased percentage of OCT4‐GFP positive cells by glutamine withdrawal in Huh7 cells. We also analyzed the correlation between Rictor/mTORC2 and stem genes and GS in TCGA database. The results showed that Rictor were indeed positively correlated with the expression of stem characteristic genes OCT4, KLF4, CD133, and EpCAM and GS (Figure 4E). Moreover, phosphorylation levels of AKT at Ser473, an indicator of mTORC2 activation,^[^ ^31^ ^]^ were positively correlated with GS and OCT4 levels, in particular when glutamine was deprived (Figure 4F). Furthermore, Rictor silencing with small hairpin RNA efficiently inhibited the phosphorylation of AKT at Ser473 and markedly prevented the upregulation of GS in response to glutamine withdrawal (Figure 4G). Rictor knock‐down significantly reduced frequency of spheroids initiating cells and spheroids formation upon glutamine starvation in vitro (Figure 4H,I; Figure S5A,B, Supporting Information). Moreover, silencing of Rictor in Huh7 cells led to retarded tumor formation and tumorigenic cell frequency (Figure 4J; Figure S5C,D, Supporting Information). GS overexpression rescued the tumor formation of Rictor‐silenced cells in vivo (Figure 4J, bottom), suggesting that mTORC2 should be the upstream of GS‐mediated stemness. These results suggest that Rictor/mTORC2 promotes GS mediated endogenous synthesis in response to exogenous glutamine deficiency, thereby regulating intracellular glutamine balance to sustain stemness of liver cancer cells.

### 2.5 Rictor was Significantly Correlated with the Expression of GS and Stem Marker OCT4 at Tumor Site, and Closely Correlated with Poor Prognosis of Liver Cancer

**Figure 5:**
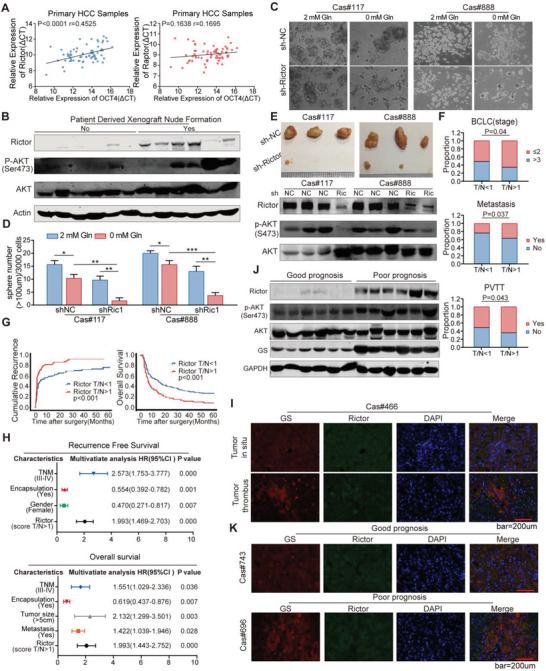
Rictor was significantly correlated with the expression of GS and stem maker OCT4 at tumor site, and closely correlated with poor prognosis of liver cancer. A) The correlation between OCT4 and Rictor (left) or Raptor (right) mRNA levels detected by Real‐Time PCR (RT‐PCR) in 69 HCC samples. B) Immunoblot assay of Rictor, AKT and phosphorylated AKT (S473) expression in fresh patient samples which can or cannot generate PDX models in nude mice. C,D) Sphere formation assay of primary patient liver cancer cells infected with lentivirus containing sh‐NC or sh‐Rictor, cultured in medium with or without glutamine for 12 days (*n* = 6). E) Xenograft tumors of primary patient liver cancer cells infected with lentivirus containing sh‐NC or sh‐Rictor; mice were injected with 1.0 × 10^6^ cells and fed for 30 days (*n* = 3); immunoblot assay detected the expression of phosphorylated AKT(S473) in each xenograft tumors. F) Kaplan–Meier analysis of the association between T (tumor)/N (para‐tumor) score of Rictor expression and multiple clinic pathological characteristics of aggressive HCC, such as high Barcelona Clinic Liver Cancer (BCLC) stage, portal vein tumor thrombus (PVTT), and metastasis in 250 primary HCC patients. G) Kaplan–Meier analysis of the association between T/N score of Rictor expression and recurrence‐free survival (RFS) or overall survival (OS) in 250 primary HCC patients. H) Multivariate analysis of hazard ratios (HRs) for tumor recurrence and OS showing that the T/N score of Rictor is an independent risk factor for RFS and OS in HCC patients. I) Representative IF staining of Rictor and GS in tissues from tumor in situ compared with tissues from tumor thrombus. Scale bar, 200 µm. J) Immunoblot analysis of Rictor, phosphorylated AKT (Ser473) and GS expression in HCC tissues from patients with poor prognosis compared with good prognosis. K) Representative IF staining of Rictor and GS in tissues from tumor in patients with good prognosis compared with poor prognosis. Scale bar, 200 µm. Spearman's correlation was used to assess the relationship between genes in (A). The data are shown in (D) as the mean values ± SD, *p* values are based on Student's *t* test. ***p < 0.001, **p < 0.01, *p < 0.05.

We further analyzed 69 matched HCC‐normal pairs of tissue using RT(real time)‐PCR. A correlation analysis between Raptor/Rictor and the stemness marker OCT4 further revealed that Rictor instead of Raptor was closely correlated with OCT4 expression (**Figure** **5**A). This supported the finding that mTORC2 rather than mTORC1 was more relative to stemness of HCC. To evaluate the clinical relevance of Rictor/mTORC2, we collected freshly resected tumors from 20 patients with HCC and generated PDX models. Among these fresh tumors, only 6 tumor samples developed primary and secondary xenograft tumors in nude mice. Remarkably, all xenograft forming tumor samples had higher levels of Rictor expression and Akt Ser473 phosphorylation than those tumor samples failed to form tumor xenografts (Figure 5B). Moreover, Rictor knock‐down in HCC cells from the secondary xenograft HCC tumors significantly reduced sphere formation capacity and prevented tumor formation in vivo (Figure 5C–E). IHC analysis of a tissue microarray (TMA) that contained 250 pairs of HCC samples was performed, and the Rictor expression was varied within these patients (Table S1, Supporting Information). Those with higher Rictor expression in tumor tissues than in adjacent normal tissues (scan score T (tumor)/N (para‐tumor)>1: *n* = 98) were associated with multiple clinic pathological characteristics of aggressive HCC such as high BCLC stage and portal vein tumor thrombus (PVTT). Moreover, metastasis (Figure 5F; Table S1, Supporting Information) and T/N score were closely correlated with poor prognosis and early recurrence in HCC patients (Figure 5G) with higher Rictor expression in cancer tissues compared with their adjacent normal tissues (scan score T/N>1: *n* = 98). The multivariate Cox regression analysis further indicated that the Rictor expression that scored T > N (Hazard ratio = 2.010; 95% confidence interval:1.483–2.724; *p* < 0.001), as well as gender, encapsulation and tumor tumor‐node‐metastasis (TNM) stage, were all independent risk factors for overall survival of HCC patients (Figure 5H; Table S2, Supporting Information). Immunofluorescence staining showed that the expression of Rictor was consistent with that of GS, and was higher in PVTT than that in situ tumor tissues (Figure 5I and Figure S5E). Moreover, western‐blotting and immunofluorescence staining showed that Rictor‐activated AKT (Ser473) signaling and GS overexpression were well correlated with poor prognosis of HCC cases (Figure 5J,K; Figure S5F, Supporting Information). Therefore, these data demonstrate that both mTORC2 activation and GS overexpression at tumor site are poor prognosis markers of HCC.

### 2.6 Rictor/mTORC2 Stabilizes GS Expression through HDAC3 Mediated Deacetylation in Response to Glutamine Starvation

**Figure 6:**
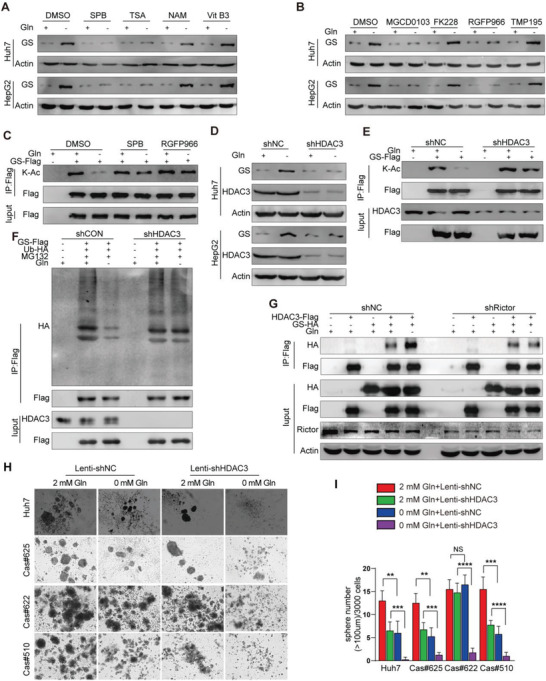
Rictor/mTORC2 stabilizes GS expression and sustains stemness of liver cancer cells through HDAC3 mediated deacetylation upon glutamine starvation. A,B) Immunoblot analysis of GS expression in Huh7 and HepG2 cells treated by different deacetylase inhibitors. Cells were separate treated with SPB (Sodium Phenylbutyrate), TSA, NAM, Vit B3, MGCD0103, FK228, RGFP966, TMP195 in the absence or presence of glutamine. C) Immunoblot analysis of acetylation levels of GS treated with SPB and RGFP966 in Huh7 cells. GS‐Flag was ectopically expressed in Huh7 cells treated with SPB or RGFP966 in the absence or presence of glutamine for 6 h, acetylation levels of flag‐bead‐purified GS were determined by K‐Ac antibody. D) Immunoblot analysis of GS expression induced by glutamine deprivation in Huh7 and HepG2 cells stably expressing sh‐NC or sh‐HDAC3 treated with or without glutamine for 6 h. E) Immunoblot analysis of acetylation levels of GS treated by glutamine deprivation in Huh7 cells stably expressing sh‐NC or sh‐HDAC3 with or without glutamine for 6 h. F) Immunoblot analysis of ubiquitination levels of GS upon glutamine deprivation treatment in control and HDAC3 knockdown cells.GS‐Flag and Ub‐HA were co‐expressed in Huh7 cells which were stably expressing shRNA against negative control and HDAC3, treated with or without glutamine for 6 h. GS was purified by immunoprecipitation (IP). Ubiquitination level of GS was probed by anti‐HA antibody. G) Immunoblot analysis of the interaction between GS and HDAC3 induced by glutamine deprivation in Rictor knockdown cells. GS‐Flag was coexpressed with HDAC3‐HA in Huh7 cells which were stably expressing shRNA against negative control and Rictor, and glutamine treatment was indicated. H,I) Sphere formation assay of Huh7 cells and primary patient liver cancer cells which infected with lentivirus containing sh‐NC or sh‐HDAC3 cultured in medium with or without glutamine for 12 days (*n* = 6). All data are shown as the mean values ± SD, *p* values are based on Student's *t* test. ****p < 0.0001, ***p < 0.001, **p < 0.01; NS, nonsignificant.

Next, to examine how mTORC2 could regulate GS expression level, we found that glutamine deprivation resulted in GS overexpression without noticeably upregulated GS mRNA levels in liver cancer cell lines (Figure S6A, Supporting Information). Glutamine was concentration dependent on GS expression in the concentration range of 0–2 × 10^−3^ m (Figure S6B, Supporting Information), while glutamine deficiency for 1 h promoted GS expression (Figure S6C, Supporting Information). Moreover, blocking protein translation with cycloheximide (CHX) did not prevented glutamine to downregulate GS (Figure S6D, Supporting Information). These results indicated that GS expression levels were regulated by glutamine at posttranslational levels. It has been reported that glutamine promotes GS degradation through increasing GS lysines11 and lysine14 acetylation,^[^ ^12^ ^]^ thus we investigated whether glutamine deficiency could stabilize GS expression by enhancing GS deacetylation. As expected, glutamine deprivation greatly decreased the acetylation and polyubiquitination levels of GS, which could be largely reversed by pan‐deacetylases inhibitor trichostatin A (TSA) and nicotinamide (NAM) treatment in Huh7 cells (Figure S6E,F, Supporting Information). Then, a serial of deacetylase inhibitors was screened to pinpoint a specific deacetylase that controlled the deacetylation and protein stability of GS. Sodium phenylbutyrate (SPB) and TSA, both pan‐HDAC inhibitors (HDACi), prevented GS upregulation upon glutamine deprivation, while NAM and Vitamin B3 (Vit B3), inhibitors of sirtuins family deacetylases, failed to affect glutamine deprivation‐mediated GS increase in both Huh7 and HepG2 cells (**Figure** **6**A). Thus, HDAC(s) might be responsible for GS stabilization upon glutamine deprivation. To determine which HDAC could be the specific deacetylase for GS, Huh7 and HepG2 were treated with HDAC1/2/3/11 inhibitor MGCD0103, HDAC3‐specific inhibitor RGFP966, HDAC1/2 inhibitor FK228, or selective class IIa HDACi TMP195. Both MGCD0103 and RGFP966 significantly decreased GS expression, while FK228 and TMP195 had no impact on GS levels (Figure 6B), indicating HDAC3 as a deacetylase of GS. This notion was further supported by RGFP966 and SPB treatment reversed the decreased acetylation level of GS mediated by glutamine withdrawal (Figure 6C). Moreover, when HDAC3 was knockdown in liver cancer cells, glutamine deprivation did not increase the protein level but did increase the acetylation and polyubiquitination of GS (Figure 6D–F), suggesting that HDAC3 as a major deacetylase determined the deacetylation and stability of GS. According to co‐IP assay, HDAC3 had the direct interaction with GS and Rictor, while GS could not interact with mTOR or Rictor, but only can interact with HDAC3 especially in the absence of glutamine (Figure S7A–D, Supporting Information). The enhanced interaction between GS and HDAC3 upon glutamine deprivation could be severely blocked by AZD2014 treatment or Rictor knockdown (Figure S7E,G, Supporting Information), indicating that the deacetylase activity of HDAC3 on GS is dependent on Rictor/mTORC2. More notably, HDAC3 knock‐down also significantly inhibited sphere formation of Huh7 cell line and primary liver cancer cells upon glutamine starvation (Figure 6H,I), suggesting that HDAC3 activity is critical to regulate the self‐renewal of TICs under glutamine starvation.

### 2.7 Blocking Rictor/mTORC2‐HDAC3/GS Inhibits Glutamine Starvation Induced Liver TICs and Promotes Tumor Regression

**Figure 7:**
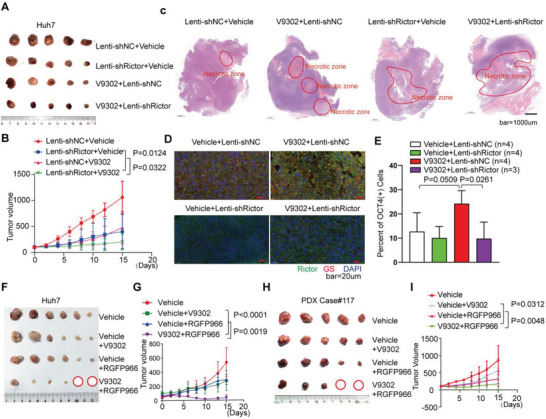
Blocking Rictor/mTORC2‐HDAC3/GS inhibits glutamine starvation induced liver TICs and promotes tumor regression. A,B) Therapeutic effect assay of shRictor‐expressing lentivirus treatment combined with V9302 in nude mice (*n* = 5). The mice were injected with 1.0 × 10^6^ Huh7 cells. After the tumor volumes were about 100 mm^3^, mice were treated with Vehicle, V9302 (75 mg kg^−1^ day^−1^), shNC‐expressing or shRictor‐expressing lentivirus (intratumoral injection of 50 µL, 5×10^7^ IU mL^−1^, rejected after 1 week) or combined treatment, and the tumor volumes were calculated every two days until the end of the experiment. C) H&E staining shows the morphology of the whole tumor tissue under different treatments. D) IF shows the expression of Rictor, GS under different treatments. E) Percent of OCT4‐GFP (+) cells detected by FC in in different groups as shown in the figure (*p* values are based on Student's *t* test). F,G) Therapeutic effect assay of V9302 combined with RGFP966 in nude mice (*n* = 6). The mice were injected with 1.0 × 10^6^ Huh7 cells. After the tumor volume were about 100 mm^3^, mice were treated with Vehicle, V9302 (75 mg kg^−1^ day^−1^), RGFP966 (10 mg kg^−1^ day^−1^) or V9302 (75 mg kg^−1^ day^−1^) and RGFP966 (10 mg kg^−1^ day^−1^) by intraperitoneal injection and the tumor volumes were calculated every other day until the end of the experiment. H,I) Therapeutic effect assay of V9302 combined with RGFP966 in PDX‐NSG mice (*n* = 5). After the tumor volume were about 100 mm^3^, mice were treated with Vehicle, V9302 (75 mg kg^−1^ day^−1^), RGFP966 (10 mg kg^−1^ day^−1^) or V9302 (75 mg kg^−1^ day^−1^) and RGFP966 (10 mg kg^−1^ day^−1^) by intraperitoneal injection and the tumor volumes were calculated every other day until the end of the experiment. The *p* value on day 15 was determined by Student's *t*‐test.

Further study, we explored the effects of targeting Rictor‐HDAC3 axis on the liver TICs and tumor growth in vivo. The results showed that blockade of glutamine uptake using V9302^[^ ^32^ ^]^ combined mTORC1/2 inhibitor AZD2014 blocking the interaction between HDAC3 and GS (Figure S7E, Supporting Information) not only significantly delayed the tumor growth but also resulted in partial or even complete tumor regression (Figure S8A–D, Supporting Information). We further verified the importance of targeting Rictor/mTORC2 for tumor starvation therapy by using shRictor‐expressing lentivirus. We found that intra‐tumoral injection of shRictor‐expressing lentivirus combined with glutamine uptake inhibitor V9302 significantly inhibited the growth of the tumor (**Figure** **7**A,B and Figure S8E,F, Supporting Information), and the tumor showed significant local necrosis (Figure 7C). In the remaining tissues, the expression of Rictor, GS and OCT4 decreased significantly in the shRictor‐expressing lentivirus combined with V9302 treatment group (Figure 7D,E). These results support the conclusion that specific inhibition of Rictor/mTORC2 inhibits TICs and promotes tumor regression under glutamine starvation. Furthermore, either HDAC3 specific inhibitor RGFP966 or glutamine uptake inhibitor V9302 delayed the tumor growth, and the combination of RGFP966 and V9302 led the tumor partial or complete regression in vivo (Figure 7F–I). Meanwhile, RGFP966 markedly prevented V9302 induced GS upregulation and inhibited the TICs in vivo (Figure S8G,H, Supporting Information). Because Rictor/mTORC2 is crucial for the interaction of HDAC3 with GS in glutamine deficiency (Figure 6G), targeting Rictor/mTORC2‐HDAC3/GS axis may effectively inhibit the characteristics of TICs in glutamine deficient microenvironment and improve the efficacy of “glutamine starvation” therapy.

## 3 Discussion

TICs have been regarded as the key factor for tumor recurrence and metastasis, which is closely related with poor prognosis in patients^[^ ^15^, ^16^ ^]^ Therefore, to deeply understand the mechanism in maintaining the stemness of TICs will greatly contribute to the development of TICs‐targeting therapeutic approach, and accordingly improve overall survival of patients. In this study, based on patient samples, PDCs and cell lines, we found that liver cancer cells relied on GS to supply glutamine and maintained stem‐like characteristics upon glutamine shortage. From a view of mechanism, glutamine shortage could activate Rictor/mTORC2 to promote HDAC3‐mediated deacetylation of GS. Moreover, both mTORC2 activation and GS overexpression were negatively related with prognosis of HCCs. Therefore, our data supply a novel mechanism of sustaining the TICs stemness, and also discover new predictive biomarkers for HCC prognosis.

Targeting glutamine transporter SLC1A5 in breast and colon cancer can inhibit the exogenous uptake of glutamine and serve the purpose of “starving” tumor cells.^[^ ^32^ ^]^ But our findings suggest that blockade of glutamine uptake would not be sufficient to starve cancer cells of glutamine, since glutamine starvation can activate GS‐mediated glutamine biosynthesis, which is conducive to stemness maintenance of cancer cells. Moreover, we found that glutamine deprivation led to the increased proportions of TICs in cancer cell population (Figure 1F–J), suggesting that glutamine starvation may even exaggerate HCC in the long run.

GS is involved in many biological processes and can regulate its own activity through post‐translational modification. GS can undergo palmitoylation to regulate the movement and migration of endothelial cells, thus affecting angiogenesis and having no direct relationship with glutamine synthesis.^[^ ^33^ ^]^ GS takes part in regulating the differentiation of Schwann cells and is regulated by ubiquitination as the substrate of E3 ubiquitin ligase ZNRF1.^[^ ^34^ ^]^ GS can also be acetylated and recognized by E3 ubiquitin ligase CRBN and the acetylation modification of the 11th and 14th lysine can be recognized by CRBN, thus promoting GS proteasomal degradation.^[^ ^11^, ^12^ ^]^ We found that glutamine deficiency induced HDAC3‐mediated GS deacetylation rendered GS resistant to degradation (Figure 6D–F). Because glutamine starvation prevents GS degradation through enhancing HDAC3‐GS interaction, ways to weaken HDAC3‐GS interaction could be a direction to develop cancer inhibiting strategies.

mTOR is known as the core receptor of intracellular nutrition, stress and energy state.^[^ ^35^, ^36^ ^]^ The anabolic processes such as glutamine synthesis are synchronized by the mammalian target of rapamycin (mTOR), which exists in two forms, mTORC1 and mTORC2, to regulate cell survival and growth in response to nutrient availability.^[^ ^37^ ^]^ Targeting mTORC1 has been shown to suppress tumor initiating cells and steer cancer initiation and metastasis.^[^ ^38^, ^39^ ^]^ But it remains unknown the role of mTOR in regulating cell survival and proliferation, especially in the face of glutamine deficiency. Glutamine has been found to activate mTORC1, regulate translation and autophagy to coordinate cell growth and proliferation.^[^ ^29^, ^40^ ^]^ While Rictor, a key component of the mTORC2 complex,^[^ ^41^ ^]^ directly phosphorylates AKT/PKB at Ser473,^[^ ^31^ ^]^ which is required for cell survival and tumor progression in various cancers^[^ ^42^, ^43^ ^]^ and suggests that mTORC2 are also involved in the regulation of cancer biology. We found that glutamine deficiency inhibited mTORC1 and activated mTORC2 signaling in hepatoma cells. Down‐regulating the expression of Rictor, a regulatory subunit of the mTORC2 complex, inhibited the increase of GS protein level induced by glutamine deficiency (Figure 4G). Our results demonstrate that mTORC2 is inversely regulated in relation to mTORC1 by glutamine deprivation for the first time. Nevertheless, inactivating mTORC2 poses a possible intervening strategy to decrease the stemness of cancer cells. Albeit we observed that Rictor could activate HDAC3 in a glutamine responsive manner (Figure 6G), the specific mechanism of how Rictor modulates the deacetylation activity of HDAC3 needs further investigation.

In conclusion, we present the first evidence that GS‐mediated endogenous glutamine synthesis is essential for maintaining and promoting the stemness of liver cancer cells both in vitro and in vivo, and provide novel insights into the role of Rictor/mTORC2‐HDAC3 in reprogramming glutamine metabolism. Targeting Rictor/mTORC2‐HDAC3 dependent glutamine synthesis may enable inhibition of TICs during glutamine starvation in vivo. It will be of great value to develop a currently unavailable Rictor/mTORC2‐specific inhibitor to inhibit TICs during nutrient restriction. More importantly, our findings suggest that the combination of Rictor/mTORC2‐HDAC3 inhibitors and glutamine uptake inhibitors might be an effective therapeutic strategy to retard tumor growth and prevent progression in patients with advanced malignant tumors.

## 4 Experimental Section

### Study Approval

All the human tissue specimens used in this study were obtained from patients who had undergone curative surgery for HCC. All subjects in our study provided written informed consent. The Ethics Committee of Huashan Hospital affiliated to Fudan University approved all human sample collection and analysis procedures (2018‐186). The experimental animal ethics committee of Fudan University approved all animal experimental procedures and protocols (JS‐092).

### Cell Culture, Plasmids, and Transfection

HEK293T, Huh7, HepG2, Hep3B, Hep1‐6 cell lines were obtained from American Type Culture Collection (ATCC). The cells were cultured in Dulbecco's modified Eagle's medium (DMEM, 4.5 g L^−1^ glucose, with or without 2 × 10^−3^ m glutamine (Gibco) supplemented with penicillin and streptomycin (Gibco), 10% fetal calf serum (FBS, Gibco). Full‐length human GS, HDAC3 were cloned into Flag or HA tagged vectors (pcDNA3‐Flag and pcDNA3‐HA). Plasmid transfection was carried out either by polyethylenimine (Merck) for HEK293T cells or lipofectamine 3000 (Invitrogen) for Huh7 and HepG2 cells.

### Primers for Knocking Down GS, Rictor, Raptor, and HDAC3

For GS, Rictor and Raptor functional study, shRNA sequences against GS, Rictor, Raptor and a scrambled negative control in a lentiviral RFP vector were employed according to the manufacturer's instructions. Titer of lentivirus containing unique shRNA was detected before transfection. The ratio of virus against cells was 5:1. At the time point of 48h following lentivirus transfection, the cells were maintained under normal cultural medium for the following experiments. Sequence (human): shRictor #1: ACCCTCTATTGCTACAATT; shRictor #2: GCAGTTACTGGTACATGAA; shRaptor #1: GCGUCACACUGGAUUUGAU shRaptor #2: CGAGAUUGGACGACCAAAU; shGS #1: ATCGTGTGTGTGAAGACTT; shGS #2: ACACCTGTAAACGGATAAT; shControl: GGGTGAACTCACGTCAGAA. Sequence (mouse): shRictor: 5′‐ GCCAGTAAGATGGGAATCA‐3′, shControl5′‐CCTAAGGTTAAGTCGCCCTCG‐3′. Lentivirus‐shHDAC3 was purchased from Santa Cruz Biotechnology(sc‐35538‐V).

### Primary Patient Liver Cancer Cells Dissociation

All the fresh tumor samples were received in the laboratory within 20 min and kept on ice. After being washed with HBSS, tumor tissues were immediately cut into small pieces of 1–2 mm and mechanically disaggregated and enzymatically digested using gentle MACSTM Dissociators and Tumor Dissociation Kit (MiltenyiBiotec, 130‐095‐929) according to the manufacturer's instructions. Then single‐cell suspensions were maintained in DMEM/F‐12 medium containing penicillin and streptomycin.

### Patient‐Derived Xenografts Animal Model

HCC tissues were obtained from therapeutic procedures performed as part of routine clinical management. The fresh human hepatoma specimens were rinsed twice with Hank's balanced salt solution (HBSS) containing antibiotic. Then tumor tissues were cut into 2 × 2 mm pieces before being implanted subcutaneously into nude mice or NOD‐scid IL2R*γ* null (NSG) mice.

### In Vivo Xenograft Assay

The nude mice, NSG mice and C57BL/6J mice aged 6–8 weeks were purchased from the Chinese Science Academy (Shanghai, China) or GemPharmatech Co., Ltd. (Jiangsu, China). Approximately 1 × 10^6 ^Huh7 cells or primary patient liver cancer cells infected by lentivirus expressing vector or shGS, shRictor, or shHDAC3 were suspended in 100 µL of DMEM and Matrigel (BD Bio‐sciences; 1:1), then they were randomly injected subcutaneously into different mice. To evaluate the effect of V9302, RGFP966 and AZD2014 treatment on tumor growth, xenograft tumors were generated with subcutaneous injections of 1 × 10^6 ^Huh7 cells or 2 × 2 mm tissue (PDX model) per animal in the right flank of nude mice or NSG mice. When the tumor grows to 100 m^3^, tumor bearing mice were randomly assigned to different treatment groups, and the indicated inhibitor treatments were performed by intraperitoneal injection for 15 days. The treatment groups consisted of vehicle, AZD2014 (10 mg kg^−1^ daily), V9302(75 mg kg^−1^ daily),^[^ ^32^ ^]^ RGPF966(10 mg kg^−1^ daily) by intraperitoneal injection. To assess the inhibition effects of shRictor‐expressing lentivirus combined V9302 on established tumors, mice received an intratumoral injection of 50 µL (5 × 10^7^ IU mL^–1^) of saline, lentivirus containing sh‐Scramble, or lentivirus containing shRictor, combined treated with or without V9302(75 mg kg^−1^ daily). After 1 week, Saline, lentivirus‐Scramble, or lentivirus‐shRictor was reinjected into the tumor mass. Tumor recordings were performed every 2 days and the mice were sacrificed at indicated time for photography. After the final treatment, the tumor was taken out for related detection.

### Statistical Analyses

Liver hepatocellular carcinoma(LIHC)data from The Cancer Genome Atlas (TCGA) cohort were analyzed. Spearman's correlation was used to assess the relationship between genes. Experimental differences between variables were assessed with two‐tailed Student's *t* test or ANOVA analysis using GraphPad Prism software (version 8.0.1). The patient's overall survival rates were calculated using the Kaplan–Meier method and log‐rank analysis. The independent factors on recurrence and survival were evaluated based on a multivariate Cox regression model. All the values were expressed as mean ±SD, statistical significance defined as *p* < 0.05; various levels of statistical significance were indicated as follows: NS, nonsignificant, *p < 0.05, **p < 0.01, ***p < 0.001, ****p < 0.0001.

## Conflict of Interest

The authors declare no conflict of interest.

## Author Contributions

H.‐L.Z., P.C., H.‐X.Y., and G.‐B.F. contributed equally to this work. H.‐L.Z., W.X., H.‐X.Y., H.‐Y.W., J.L. designed the research; H.‐L.Z., P.‐C., G.‐B.F. performed research; H.‐X.Y., S.‐M.Z. contributed in reagents/analytic tools; F.‐F.L., J.Z., J.‐H.Y., L.C., J.Z. analyzed data; H.‐Y.W., B.Z., H.‐S.C., J.C., S.H. contributed to project discussions; H.‐L.Z. and J.L. wrote the manuscript.

## Supporting information

Supporting Information

## Data Availability

The data that support the findings of this study are available from the corresponding author upon reasonable request.
