# Supplementary material for: Targeting mTORC2/HDAC3 Inhibits Stemness of Liver Cancer Cells Against Glutamine Starvation
Source: Adv Sci (Weinh). 2022 Feb 20;9(20):2103887. doi: 10.1002/advs.202103887 (PMC9284171; doi:10.1002/advs.202103887)
Supplement: Supplementary file 1 — Supporting Information [file ADVS-9-2103887-s001.pdf]

ADVANCED SCIENCE
Open Access

Supporting Information

for *Adv. Sci.*, DOI 10.1002/advs.202103887

Targeting mTORC2/HDAC3 Inhibits Stemness of Liver Cancer Cells Against Glutamine Starvation

Hui-Lu Zhang, Ping Chen, He-Xin Yan, Gong-Bo Fu, Fei-Fei Luo, Jun Zhang, Shi-Min Zhao, Bo Zhai, Jiang-Hong Yu, Lin Chen, Hao-Shu Cui, Jian Chen, Shuai Huang, Jun Zeng, Wei Xu, Hong-Yang Wang* and Jie Liu**

Supplementary experimental materials and Figures

Targeting mTORC2/HDAC3 inhibits stemness of liver cancer cells against glutamine starvation

Running Title: **Rictor/mTORC2 senses glutamine levels to regulate stemness of liver cancer cells**

Hui-Lu Zhang[1,7], Ping-Chen[1,7], He-Xin Yan[2,7], Gong-Bo Fu[5,7,] Fei-Fei Luo[1], Jun Zhang[1], Shi-Min Zhao[1], Bo Zhai[2], Jiang-Hong Yu[1], Lin Chen[1], Hao-Shu Cui[1], Jian Chen[1], Shuai Huang[2], Jun Zeng[6], Wei Xu[1]*, Hong-Yang Wang[3,4]* Jie Liu[1]*

1. Huashan Hospital of Fudan University and Institutes of Biomedical Sciences, Shanghai 200040, China;
2. Renji Hospital, School of Medicine, Shanghai Jiaotong University, Shanghai 200120, China;
3. Eastern Hepatobiliary Surgery Hospital, Second Military Medical University, Shanghai 200433, China.
4. National Center for Liver Cancer, Shanghai 200433, China;
5. Department of Medical Oncology, Affiliated Jinling Hospital, Medical School of Nanjing University, Nanjing 210093, China
6. Key Laboratory of Separation Science for Analytical Chemistry, Dalian Institute of Chemical Physics, Chinese Academy of Sciences, Dalian 116023, China
7. These authors contributed equally to this work.

Conflict of Interest: The authors declare no conflict of interest

*Correspondence: jieliu@fudan.edu.cn(Jie Liu), hywangk@vip.sina.com(Hong-Yang Wang), xuwei_0706@fudan.edu.cn(Wei Xu)

Supplementary materials and methods

Antibodies and Reagents Antibodies specific to CD133 (Abcam, ab19898), EpCAM (Abcam, ab223582), OCT4A (Cell Signaling technology, #2890), Sox2(Cell Signaling technology, #14962), ATF4(proteintech, 10835-1-AP),ASCT2(proteintech,20350-1-AP),SLC7A5(proteintech,28670-1-AP)KLF4(proteintech, 11880-1-AP), Rictor (Abcam, ab104838; proteintech,27248-1-AP), GS(Santa Cruz Biotechnology, sc-74430;proteintech, 66323-1-Ig), phospho-p70 S6 Kinase (Thr389) (Cell Signaling technology, #9234), AKT(Cell Signaling technology, #4685), phosphor-AKT(Ser473)(Cell Signaling technology, #4060), phospho-4EBP1(Thr37/46)(Cell Signaling technology, #2855); HDAC3(Santa Cruz Biotechnology , sc-376957), HA (Abmart, M20003), Flag (Abmart, M20008), GAPDH (Cell Signaling technology, #5174) and beta-Actin (GenScript, A00702) were purchased commercially. The generation of pan-Acetyl-Lysine antibody was described previously[1]. L-MSO(M5379), BPTES (SML0601), Dimethyl a-ketoglutarate(dm-aKG)(#349631) and Neucleotides (K10025A) was obtained from Sigma Aldrich. Rapamycin(S1039), AZD2014(S2783), Trichostatin A (TSA) (S1045) and Nicotinamide(S1899), V9302(S8818), Sodium Phenylbutyrate (SPB)(S4125), MGCD0103(S1122), RGFP966(S7229), FK228(S3020), TMP195(S8502) were purchased from Selleck.

Cell proliferation and sphere formation assay

Cell viabilities were monitored by the cholecystokinin-8 (CCK-8) (Dojindo Laboratories, Kumamoto, Japan) assay. Isolated primary hepatoma cells and routine cultured HCC cell lines were seeded in triplicates in 100 μl of complete medium at a density of 5000 viable cells per well in 96-well plates. Following an incubation overnight, the cells were washed with PBS before being maintained in complete medium with or without additional indicated reagents. For glutamine deprivation experiments, the cells were washed with PBS and then starved in 100μl glutamine-free Dulbecco’s modified Eagle’s medium supplemented with 5%FBS. At the time points indicated, each well was mixed with 10 μl CCK-8 before being incubated for an additional 1 h before OD values were detected at an absorbance of 450 nm using a microplate reader (Synergy HT, USA). Cell viability was presented as a proportion of the control value. For cell sphere formation assay, routine cultured HCC cell lines or isolated primary hepatoma cells were plated in Ultra-low attachment well (CORNING) culturing with medium without serum, to be kept there for 12 days. The sphere was counted, diameter greater than 100um.

Real-time PCR

Total RNA of cultured cells or human HCC samples were extracted using TRIzol reagent (Invitrogen) according to the manufacturer’s protocols. Real-time PCR analyses were performed using an ABI 7300 Fast Real-Time PCR System (Applied Biosystems, Foster City, CA) and SYBR Green PCR kit (Applied TaKaRa, Otsu, Shiga, Japan). The ΔΔCt method was used with actin as an endogenous control for normalization of the results. The following primers were purchased from Invitrogen: (1) β-actin: forward 5'-GACTACCTCATGAAGATC-3', reverse 5'-GATCCACATCTGCTGGAA-3'; (2) ATF4: forward5'- GGCCACCATGGCGTATTA -3', reverse 5'- TGCTGAATGCCGTGAGAA-3'; (3)

OCT4:forward5'-AGCTTCCAAGGCCCTCC-3',reverse5'-CTCCTCCGGGTTTTGCTCC-3';(4) SOX2: forward 5'-CAAAAATGGCCATGCAGGTT-3', reverse 5'-AGTTGGGATCGAACAAAAGCTATT-3';(5) KLF4:forward5'-GTCCCGGGGATTTGTAGCTC-3', reverse5'-TGTAGTGCTTTCTGGCTGGG-3'.(6) SLC1A5: forward 5'-CAACCTGGTGTCAGCAGCCTT-3', reverse5'- GCACCGTCCATGTTGACGGTG-3'; (7) SLC7A5: forward 5'- AAATGATCAACCCCTACAGAAACCT -3', reverse5'- ACGTACACCAGCGTC ACGAT-3';(8) GS: forward 5'- ACTCAGGGGAGCAAAGGAAG-3', reverse5'- CACACAATCTTGGCATTTCC-3';

Immunoblotting and Immunoprecipitation

Protein extracts were prepared in lysis buffer, and western blot analysis was performed as described previously. Briefly, cultured cells or liver cancer tissues were lysed in Triton lysis buffer (20 mM Tris, pH 7.4, 137 mM NaCl, 10% glycerol, 1% Triton X-100, 2 mM EDTA, 1 mM PMSF, 10 mM sodium fluoride, 5 mg/ml of aprotinin, 20 mM leupeptin and 1 mM sodium orthovanadate) and centrifuged at 12 000g for 15 min. Protein concentrations were determined via the BCA assay kit according to manufacturer’s protocols. After incubating with the fluorescein conjugated secondary antibody, the immunocomplexes were detected using an Odyssey fluorescence scanner (Li-Cor, Lincoln, NE). For immunoprecipitation experiments, a total of 1mg of treated HCC cell lysates were incubated with 2μg anti-IgG, specific antibodies or normal rabbit immunoglobulin G (Santa Cruz Biotechnology) for 8 hours at 4°C, followed by addition of Protein A/G Plus-Agarose (Santa Cruz Biotechnology) for another 3 hours. The samples were denatured and subjected to immunoblotting analyses.

Immunohistochemistry

Tissue microarray slides were deparaffinized and rehydrated in ethanol, and then treated with 0.3% hydrogen peroxide in methanol to block the endogenous peroxidase activity. After blocking with 2% BSA in PBS for 30 min, sections were incubated with 1:100 diluted rabbit anti-GS or anti-Rictor antibody at 4℃ overnight. Corresponding secondary antibodies were used, and diaminobenzidine (DAB) (Dako, Carpinteria, CA) staining was applied. Sections were counterstained with hematoxylin. Assessment of the immunostaining was performed using the Image-scop software (Media Cybernetics, Inc., Bethesda, MD) according to the staining intensities and the percentage of positively stained cells.

Untargeted Metabolomics analysis

Analyses were performed using an UHPLC (1290 Infinity LC, Agilent Technologies) coupled to a quadrupole time-of-flight (AB Sciex TripleTOF 6600). For HILIC separation, samples were analyzed using a 2.1 mm × 100 mm ACQUIY UPLC BEH 1.7 µm column (waters, Ireland). In both ESI positive and negative modes, the mobile phase contained A=25 mM ammonium acetate and 25 mM ammonium hydroxide in water and B= acetonitrile. The gradient was 85% B for 1 min and was linearly reduced to 65% in 11 min, and then was reduced to 40% in 0.1 min and kept for 4 min, and then increased to 85% in 0.1 min, with a 5 min re-equilibration period employed. The ESI source conditions were set as follows: Ion Source Gas1 (Gas1) as 60, Ion Source Gas2 (Gas2) as 60, curtain gas (CUR) as 30, source temperature: 600°C, IonSpray Voltage Floating (ISVF) ±

5500 V. In MS only acquisition, the instrument was set to acquire over the m/z range 60-1000 Da, and the accumulation time for TOF MS scan was set at 0.20 s/spectra. In auto MS/MS acquisition, the instrument was set to acquire over the m/z range 25-1000 Da, and the accumulation time for product ion scan was set at 0.05 s/spectra. The product ion scan is acquired using information dependent acquisition (IDA) with high sensitivity mode selected. The collision energy (CE) was fixed at 35 V with ± 15 eV. Declustering potential (DP) was set as ±60 V. The raw MS data (wiff.scan files) were converted to MzXML files using ProteoWizard MSConvert and processed using XCMS for feature detection, retention time correction and alignment. The metabolites were identified by accuracy mass (<25 ppm) and MS/MS data which were matched with our standards database. In the extracted ion features, only the variables having more than 50% of the nonzero measurement values in at least one group were kept. For the multivariate statistical analysis, the MetaboAnalyst (www.metaboanalyst.ca) web-based system was used. After the Pareto scaling, principal component analysis (PCA) and partial least-squares-discriminant analysis (PLS-DA) were performed. The leave one out cross-validation and response permutation testing were used to evaluate the robustness of the model. The significant different metabolites were determined based on the combination of a statistically significant threshold of variable influence on projection (VIP) values obtained from PLS-DA model and two-tailed Student's t test (p value) on the raw data, and the metabolites with VIP values larger than 1.0 and p values less than 0.1 were considered as significant. Metabolic pathway enrichment analysis was performed using MetaboAnalyst[2].

Supplementary figures

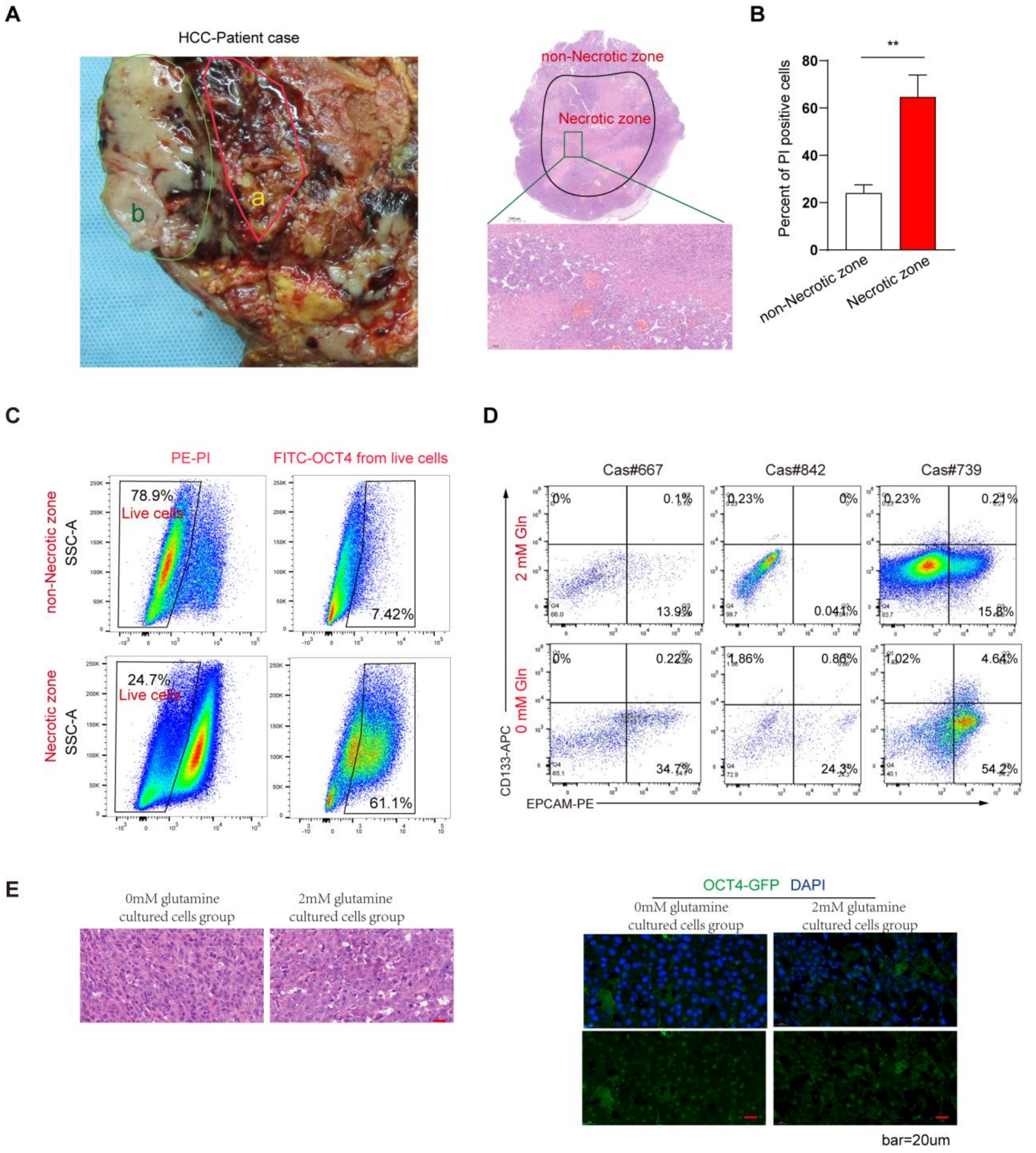

Figure S1 Liver cancer cells display stem-like characteristics upon glutamine shortage. (A) The necrotic (the area marked “a”) and non necrotic(the area marked “b”) areas of liver cancer samples were displayed; H&E staining showed more tissue necrosis and morphology in the central area of tumor tissue; (B) Cell viability assessment by propidium iodide (PI) staining in the core zones with more necrotic tissue areas and in the periphery zones with no necrotic tissue areas of fresh tumors from HCC patients (n=3); (C) Representative images of cell viability assessment by PI staining and OCT4 positive cells

detection in the core zones with more necrotic tissue areas and in the periphery zones with no necrotic tissue areas of fresh tumors; (D) Representative images of CD133 and EPCAM expression in primary patient liver cancer cells detected by FCM cultured in medium with or without glutamine for 48 hours (n=3). (E) H&E and IF staining of OCT4 in tumor tissue formed by cells treated with 0mM and 2mM glutamine.

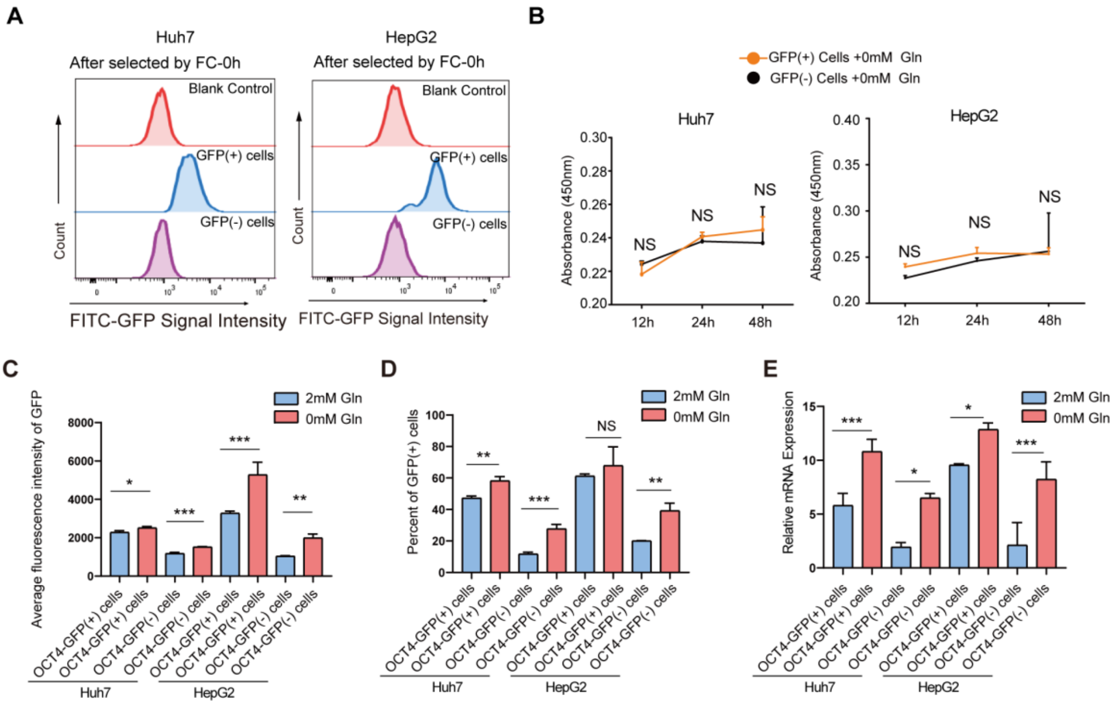

Figure S2 OCT4-GFP(+) HCC stem cells had no proliferation advantage compared with OCT4-GFP(-) HCC cells in glutamine deficiency within 48 hours. (A) OCT4-GFP (+) cells and OCT4-GFP (-) cells were separated by flow cytometry (FC). (B) GFP positive cells and GFP negative cells were sorted into 96 well plates by FC and their proliferation ability was detected by CCK8.(C-D)OCT4-GFP (+) and OCT4-GFP (-) cells were selected by FC and cultured with or without glutamine for 48 hours. The average fluorescence intensity of GFP and the proportion of GFP (+) cells were detected by FITC channel of FC (n=3). (E) OCT4-GFP (+) and OCT4-GFP (-) cells were selected by FC and cultured with or without glutamine for 48 hours. The expression of OCT4 was detected by Real Time PCR (n=3). All data are shown as the mean values ± SD, p values are based on student's t test. ****$p < 0.0001$, ***$p < 0.001$, **$p < 0.01$, *$p < 0.05$; NS, non-significant.

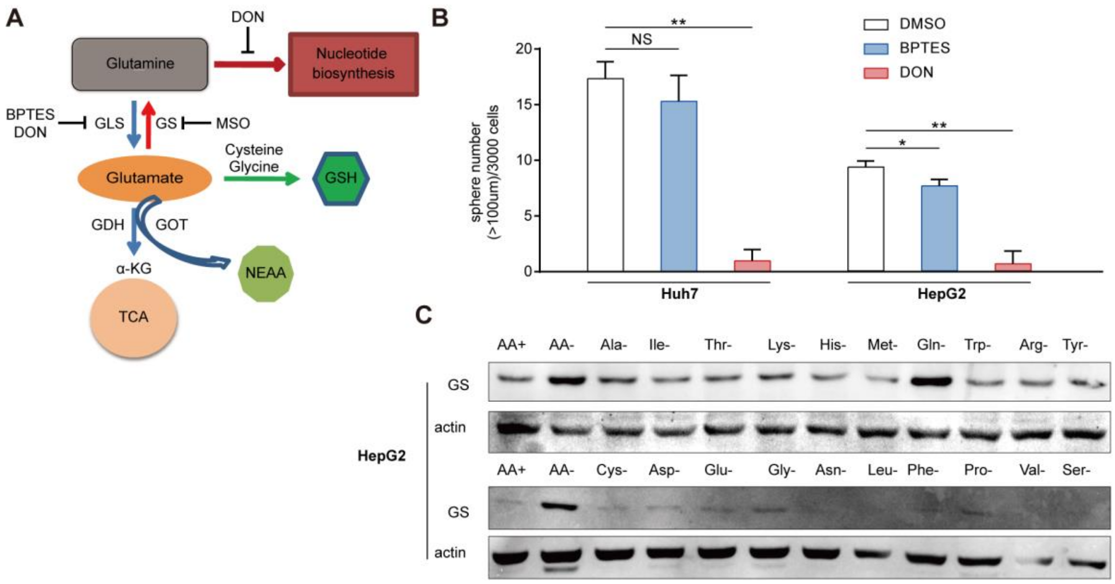

Figure S3 GS responds to glutamine deficiency and maintains stem cell self-renewal. (A) Diagram of glutamine metabolic pathways and target inhibitors. (B) Quantification of the sphere numbers in sphere formation assay of Huh7 and HepG2 cells with treatment of BPTES (10 μM) or DON (50 μM) (n=6). (C) Immunoblot assay of GS expression in HepG2 cells treated by different amino acid deprivation for 6 hours. AA represents all 20 kind of amino acids.All data are shown as the mean values ± SD, p values are based on student's t test. **p < 0.01, *p < 0.05; NS, non-significant.

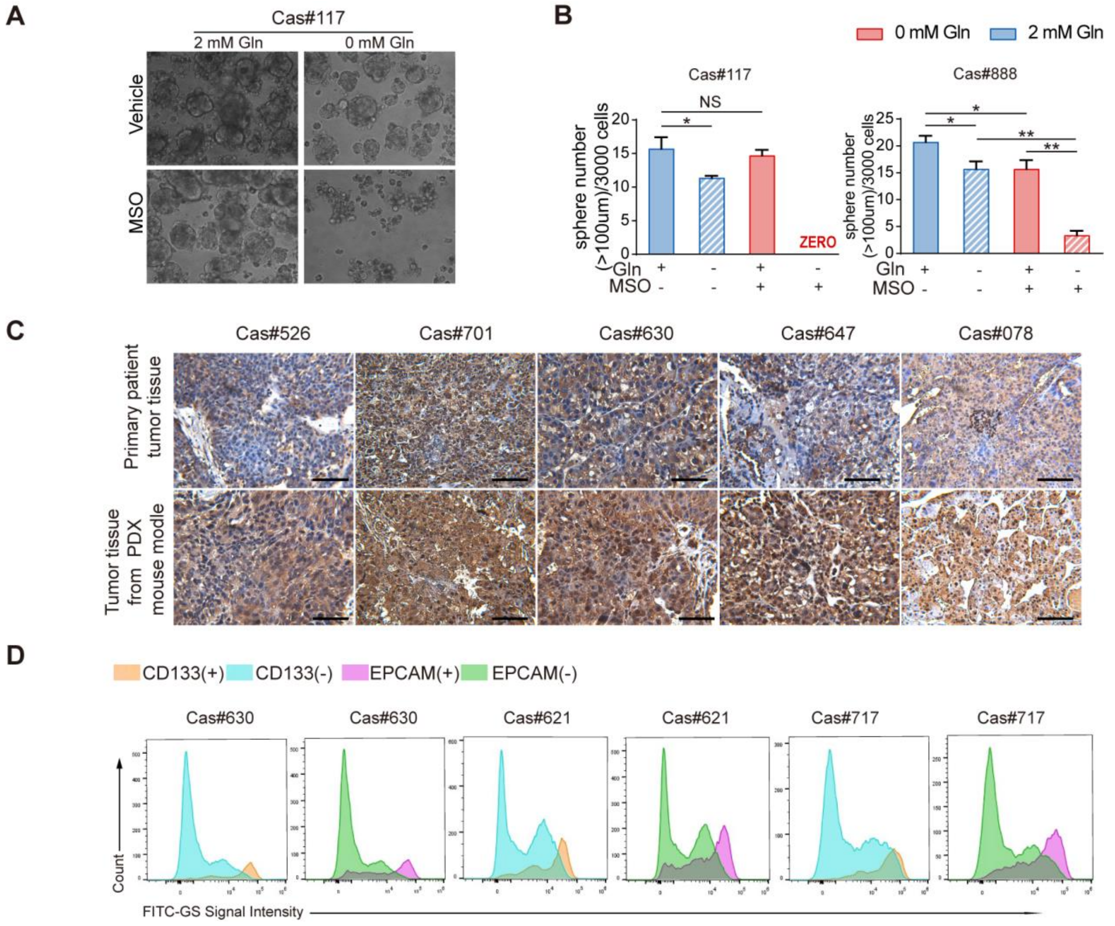

Figure S4 Clinical sample studies show that GS is highly expressed in stem cell subsets and tumor formation, and maintains the self-renewal of cancer stem cells. (A and B) Representative images(A) and quantification of the sphere numbers(B) of primary liver cancer cells with treatment of MSO (1 mM) in medium with or without glutamine (n=6). (C) Representative images of GS staining by immunohistochemistry (IHC) in tissues from primary patient tumor compared with tissues from Patient-Derived tumor Xenograft (PDX) mouse model. Scale bar, 200 μm. (D) Representative images of GS expression in CD133 and EPCAM positive or negative cells detected by FCM in primary patient liver cancer cells. Data are shown in (B) as the mean values ± SD, p values are based on student's t test. $^{**}p < 0.01$, $^{*}p < 0.05$; NS, non-significant.

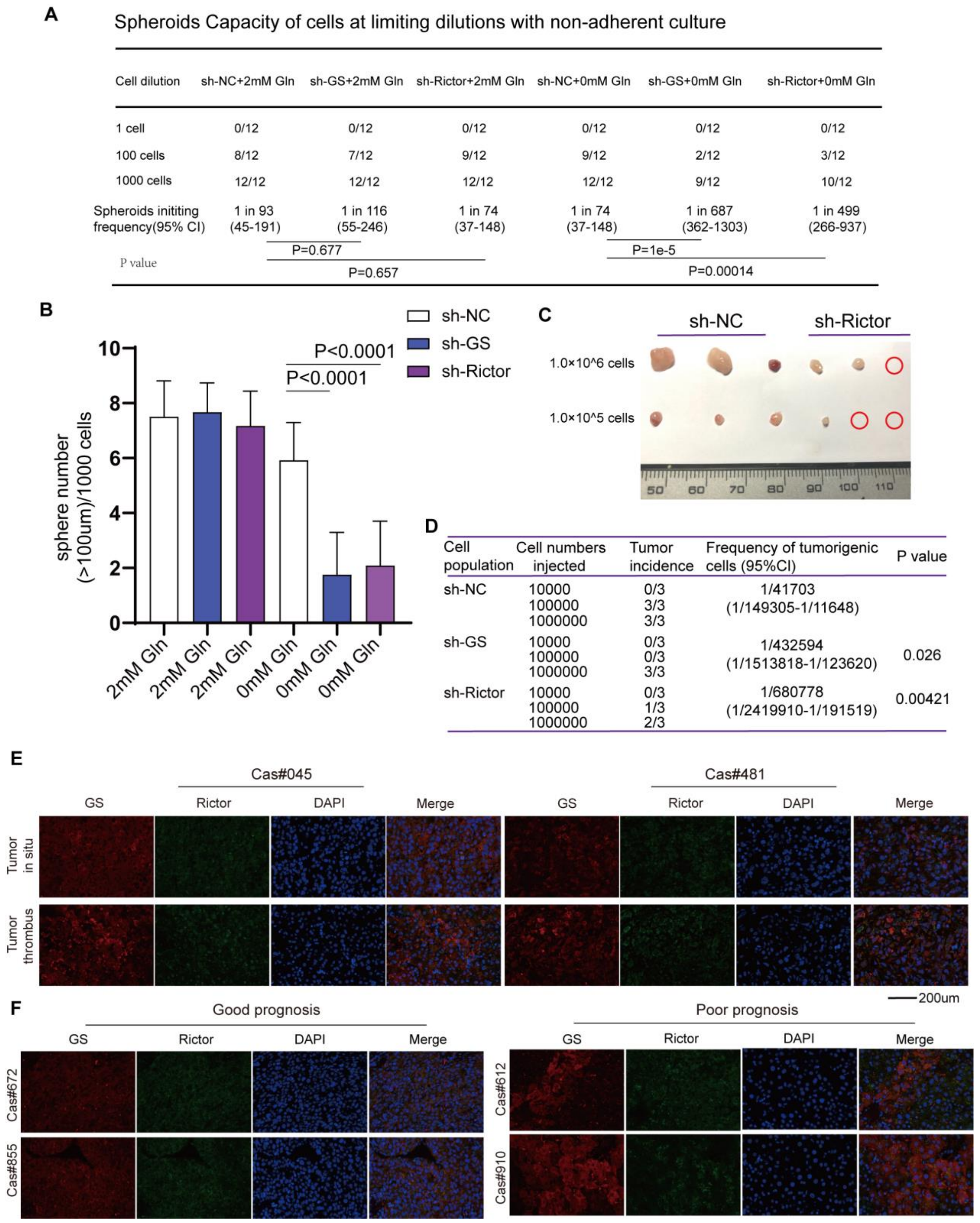

Figure S5 Rictor and GS affect the characteristics of tumor stem cells and are related to tumor prognosis (A-B) The calculation of spheroids initiating cells frequency at limiting dilutions of Huh7 cells expressing sh-NS, sh-Rictor or sh-GS with suspension culture for 12 days (n=12) (http://bioinf.wehi.edu.au/software/elda/). CI, confidence interval. (C) Representative images of xenograft tumors injected with 1.0×10^6 and 1.0×10^5 Huh7 cells expressing sh-NC and sh-Rictor in NSG mice (n=3). (D)Tumorigenic cell

frequency in GS, Rictor knockdown and negative control primary tumor cells was analyzed with a limiting dilution assay (http://bioinf.wehi.edu.au/software/elda/). CI, confidence interval. (E) Representative IF staining of Rictor and GS in tissues from tumor in situ compared with tissues from tumor thrombus. (F) Representative IF staining of Rictor and GS in tissues from tumor in patients with good prognosis compared with poor prognosis. Scale bar, 200 μm. Data are shown in (B) as the mean values ± SD, p values are based on student's t test. $^{**}p < 0.01$, $^{*}p < 0.05$; NS, non-significant.

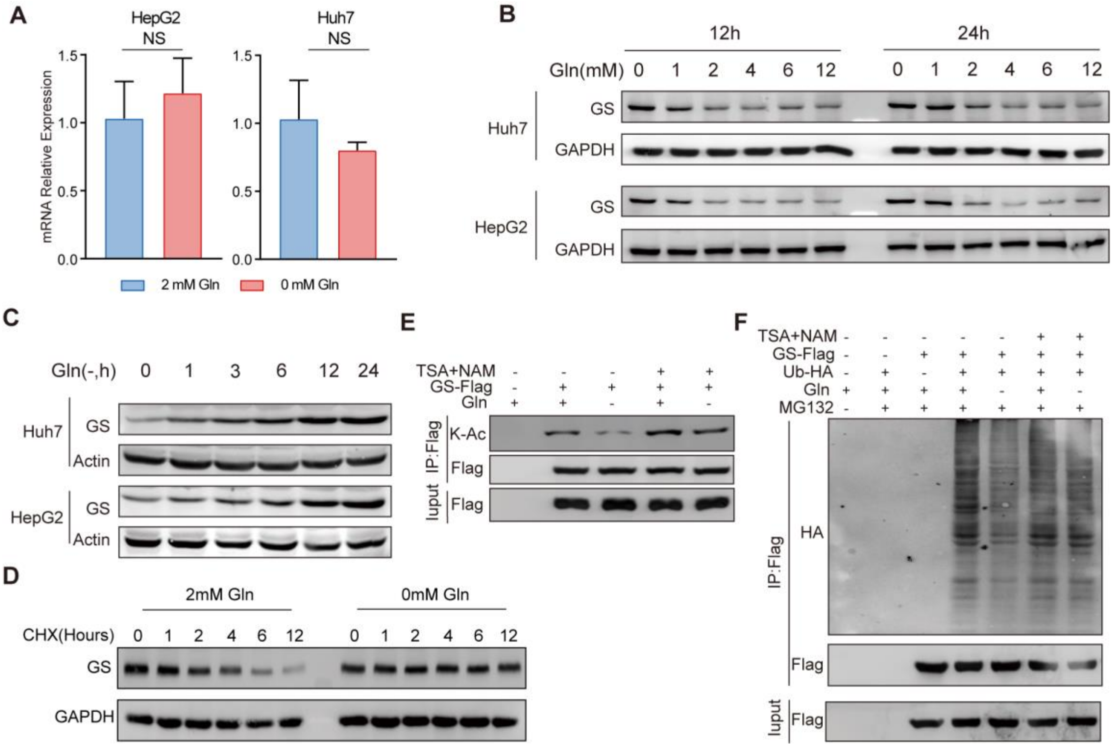

Figure S6 Glutamine deficiency could stabilize GS expression by enhancing GS deacetylation. (A) Relative mRNA levels of GS of Huh7 and HepG2 cells upon glutamine deficiency treatment. The expression of GS was analyzed by RT-PCR under glutamine starvation treatment for 12 hours. (B) Immunoblot analysis of GS expression in different concentrations of glutamine. (C) The effect of glutamine starvation at different time on GS expression was analyzed by immunoblotting. (D) Immunoblot analysis of GS degradation rates in Huh7 cells treated with(2mM) or without glutamine in the presence of 100 mg/ml cycloheximide (CHX) for the indicated times were measured. (E) Immunoblot analysis of GS acetylation levels in Huh7 cells treated with TSA and NAM in the absence or presence

of glutamine for 6 hours. Acetylation levels of Flag-bead-purified GS were determined by immunoblot analysis using a pan-anti-acetyllysine antibody (K-Ac). (F) Immunoblot analysis of GS ubiquitination levels of Huh7 cells treated with deacetylase inhibitors in the absence or presence of glutamine for 6 hours. Huh7 cells were transfected with GS-Flag and Ub-HA, under treated or not treated with 0.5 mM TSA (Trichostatin A) and 5 mM NAM (N-Acetyl-L-Methionine) in the absence or presence of glutamine for 6 hours. GS was purified by immunoprecipitation (IP). Ubiquitination level of GS was probed by anti-HA antibody.

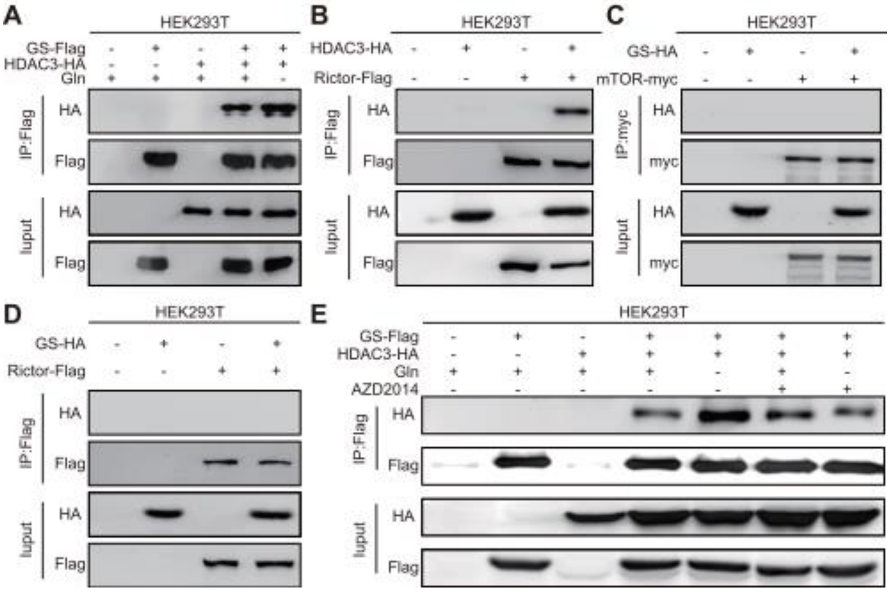

Figure S7 HDAC3 had the direct interaction with GS and Rictor. (A) Immunoblot analysis of the interaction between GS and HDAC3. Interaction between GS-Flag and HDAC3-HA co-expressed in HEK293T cells under treated or not treated with glutamine was determined. (B) Immunoblot analysis of the interaction between HDAC3 and Rictor. (C and D) Immunoblot analysis of the interaction between GS and mTOR or Rictor. (E) Immunoblot analysis of the interaction between GS and HDAC3 in HEK293T cells under treated or not treated with AZD2014 in the absence or presence of glutamine for 6 hours.

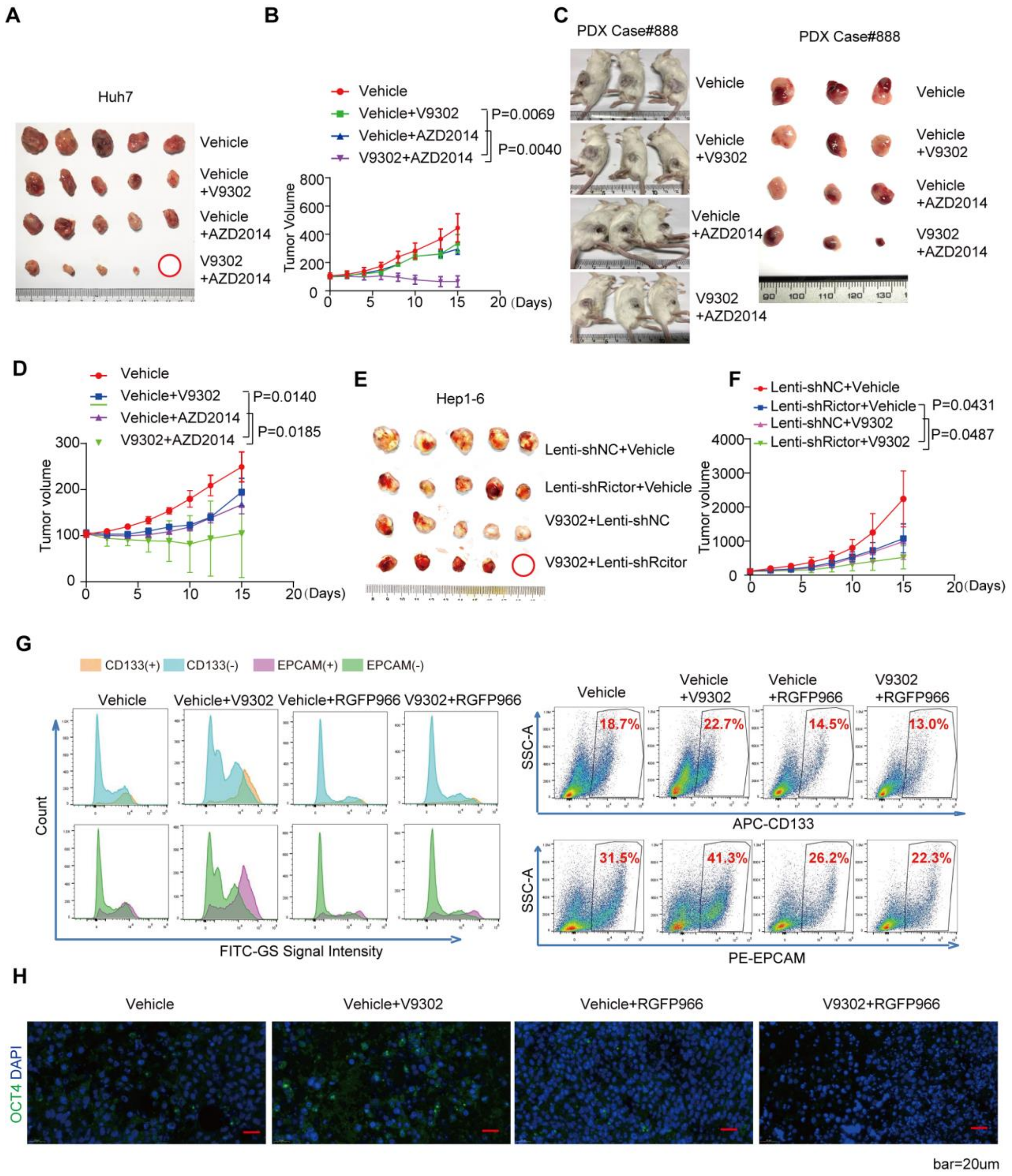

Figure S8 Blocking Rictor/mTORC2-HDAC3/GS inhibits glutamine starvation induced liver TICs and promotes tumor regression. (A-D)Therapeutic effect assay of V9302 combined with AZD2014 treatment in nude mice (n=5) and in PDX-NSG mice (n=3). The nude mice were injected with 1.0×10^6 Huh7 cells and NSG mice were injected with patient dirived tissures. After the tumor volumes were about 100 mm3, mice were treated with Vehicle, V9302 (75 mg/kg/day), AZD2014 (10 mg/kg/day) or V9302 (75 mg/kg/day) and AZD2014 (10 mg/kg/day) by intraperitoneal injection, and the tumor volumes were calculated every two days until the end of the experiment. (E-F)

Therapeutic effect assay of shRictor-expressing lentivirus treatment combined with V9302 in C57BL/6J mice (n=5). The mice were injected with 1.0×10^6 Hep1-6 cells. After the tumor volumes were about 100 mm^3, mice were treated with Vehicle, V9302 (75 mg/kg/day), shNC-expressing or shRictor-expressing lentivirus (intratumoral injection of 50 μl, 5*107 IU/ml, rejected after 1 week) or combined treatment, and the tumor volumes were calculated every two days until the end of the experiment (Day 15). (G) GS expression and percent of CD133 and EPCAM positive cells were detected by FC in Vehicle, V9302, RGFP966 or V9302 and RGFP966 treated tumors. (H) IF shows the expression of OCT4 in Vehicle, V9302, RGFP966 or V9302 and RGFP966 treated tumors. The P value on day 15 was determined by Student’s t-test.

Supplementary tables

Table S1

Supplementary Table 1.Clinico-pathological characteristics and Rictor expression

Variable*		Number and value (range)		*P* value
		RictorT/N>1 (n=98)	Rictor T/N<1 (n=152)	
Age (range. yrs)	≤50	60	80	.228*
	>50	38	72	
Gender	Male	83	137	.275
	Female	15	15	
AFP (ug/L)	≤400	28	50	.471
	>400	70	102	
Tumor size	≤5cm	15	35	.184
	>5cm	83	117	
Pathological satellite	Yes	46	69	.913
	No	52	83	
Encapsulation	Complete	37	55	.907
	No	61	97	
BCLC(stage)	≤2	33	73	.04
	>3	65	79	
PVTT	Yes	63	78	.043
	No	35	74	
Metastasis	Yes	36	36	.037
	No	62	116	

Note: P<0.05 by Chi-square test or Student t test,*:Mann-Whitney test. AFP, a-fetoprotein.

Table S2

Univariate Analyses Of Clinical Variables Correlated With Recurrence And Survival

Characteristics	OS		RFS	
	HR(95% CI)	P Value	HR(95% CI)	P Value
Gender (Female vs. Male)	0.496(0.297-0.829)	0.007	0.609(0.377-0.985)	0.043
Age (≤50 vs. >50)	0.810(0.607-1.080)	0.151	0.830(0.621-1.111)	0.210
AFP level, ng/ml (≤400 vs. >400)	1.478(1.071-2.041)	0.018	1.352(0.987-1.852)	0.061
Maximal tumor size, cm (≤5 vs. >5)	2.527(1.665-3.835)	0.000	2.844(1.868-4.329)	0.000
Tumor nodules number (Single vs. Multiple)	1.264(1.087-1.470)	0.002	1.111(0.929-1.330)	0.248
Metastasis (No vs. Yes)	1.319(0.967-1.801)	0.081	1.851(1.369-2.503)	0.000
Microvascular invasion (No vs.Yes)	2.536(1.441-4.463)	0.001	1.994(1.206-3.295)	0.007
Encapsulation (Complete vs. None)	0.463(0.334-0.640)	0.000	0.572(0.418-0.783)	0.000
BCLC Stage (Ⅰ-Ⅱ vs. Ⅲ-Ⅳ)	2.617(1.920-3.568)	0.000	1.751(1.295-2.366)	0.000
Tumor TNM stage (Ⅰ-Ⅱ vs. Ⅲ-Ⅳ)	3.026(2.098-4.362)	0.000	2.575(1.806-3.671)	0.000
Rictor (T<N vs. T>N)	1.781(1.329-2.386)	0.000	1.819(1.349-2.451)	0.000

Note:Univariate analysis was calculated by the Kaplan-Meier method,(P<0.05 by log-rank test).AFP, a-fetoprotein

1. Zhao, S. *et al.* Regulation of cellular metabolism by protein lysine acetylation. *Science* **327**, 1000-1004 (2010).

2. Xia, J., Sinelnikov, I.V., Han, B. & Wishart, D.S. MetaboAnalyst 3.0--making metabolomics more meaningful. *Nucleic Acids Res* **43**, W251-257 (2015).
